# Supplementary material for: Tuning the selectivity of catalytic nitriles hydrogenation by structure regulation in atomically dispersed Pd catalysts
Source: Nat Commun. 2021 Oct 26;12:6194. doi: 10.1038/s41467-021-26542-y (PMC8548558; doi:10.1038/s41467-021-26542-y)
Supplement: Supplementary file 1 — Supplementary Information [file 41467_2021_26542_MOESM1_ESM.pdf]

Supplementary Information

Tuning the selectivity of catalytic nitriles hydrogenation by structure regulation in atomically dispersed Pd catalysts

*Zhibo Liu[1,2 #], Fei Huang[2,3 #], Mi Peng[4 #], Yunlei Chen[5,6#], Xiangbin Cai[7], Linlin Wang [1,2], Zenan Hu[1], Xiaodong Wen[5,6], Ning Wang [7], Dequan Xiao[8], Hong Jiang[4], Hongbin Sun[1 *], Hongyang Liu[2,3 *] and Ding Ma[4 *]*

[1] Department of Chemistry, Northeastern University, Shenyang, 110819, P. R.China.

[2] Shenyang National Laboratory for Materials Science, Institute of Metal Research, Chinese Academy of Sciences, Shenyang 110016, P. R. China.

[3] School of Materials Science and Engineering, University of Science and Technology of China, Shenyang 110016, P. R. China.

[4] Beijing National Laboratory for Molecular Sciences, College of Chemistry and Molecular Engineering and College of Engineering, and BIC-ESAT, Peking University, Beijing 100871, P. R. China.

[5] State Key Laboratory of Coal Conversion, Institute Coal Chemistry, Chinese Academy of Sciences, Taiyuan 030001, P. R. China.

[6] University of Chinese Academy of Science, No. 19A Yuanquan Road, Beijing 100049, P. R. China.

[7] Department of Physics and Center for Quantum Materials, Hong Kong University of Science and Technology, Clear Water Bay, Kowloon, Hong Kong SAR, P. R. China.

[8] Center for Integrative Materials Discovery, Department of Chemistry and Chemical Engineering, University of New Haven, 300 Boston Post Road, West Haven, Connecticut 06516, United States.[*] These authors contributed equally to this work.

Corresponding Author

*Email: liuhy@imr.ac.cn; sunhb@mail.neu.edu.cn; dma@pku.edu.cn

1. Supplementary Figures and Tables

Supplementary Table 1. Physicochemical parameters of different samples.

Sample	Pd[a] [wt%]	S_{BET}[b] [m^2 g^{-1}]	V_{pore}[c] [cm^3 g^{-1}]	Pore size[d] [nm]
ND@G	-	331	1.45	17.5
Pd_1/ND@G	0.11	332	1.50	13.8
Pd_n/ND@G	1.90	345	1.38	15.4

[a] Determined by ICP-AES. [b] BET method. [c] Volume of N_2 at p/p^0 = 0.98. [d] N_2 desorption.

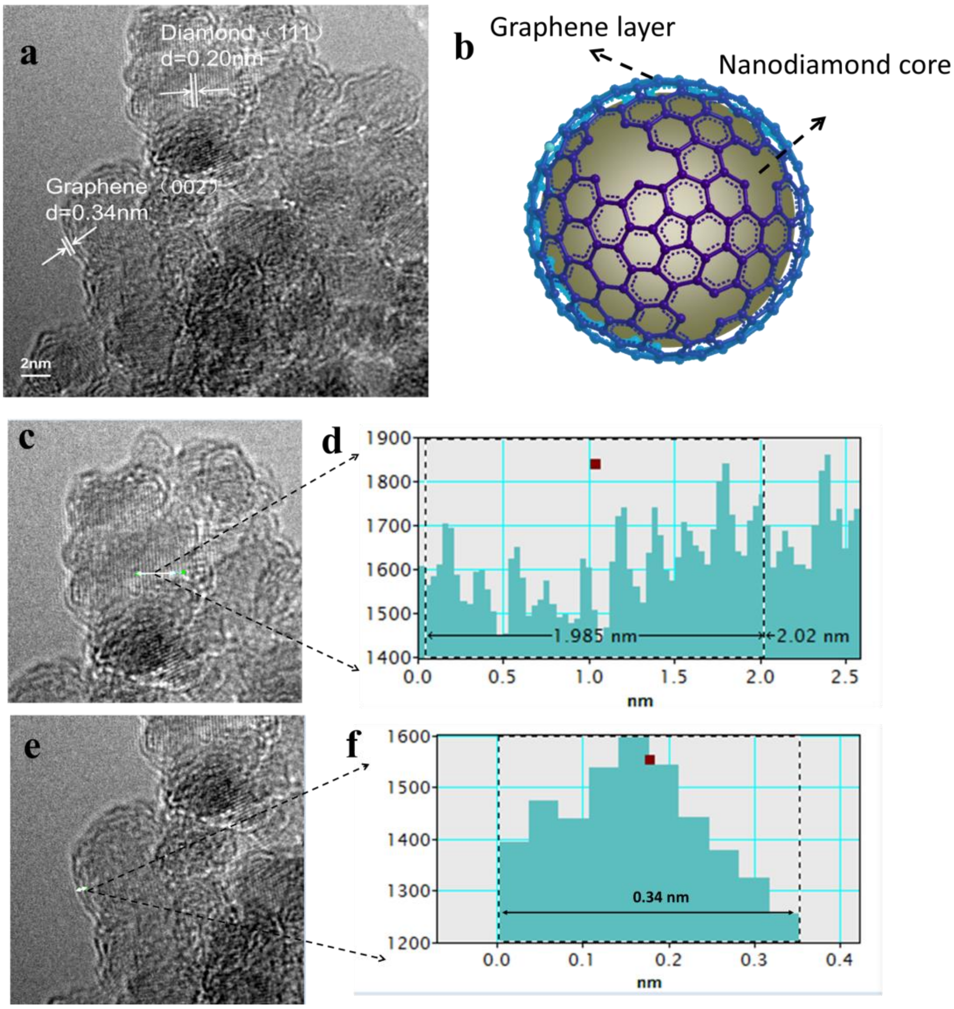

Supplementary Figure 1. (a) HRTEM image of the as-prepared ND@G support with nanodiamond core and defective graphene shell; (b) Structure diagram of ND@G; (c) Partial enlargemen HRTEM image focusing on the nanodiamond core and (e) The corresponding line profile of the nanodiamond core; (e) Partial enlargemen HRTEM image focusing on the defective graphene shell and (f) The corresponding line profile of the defective graphene shell.

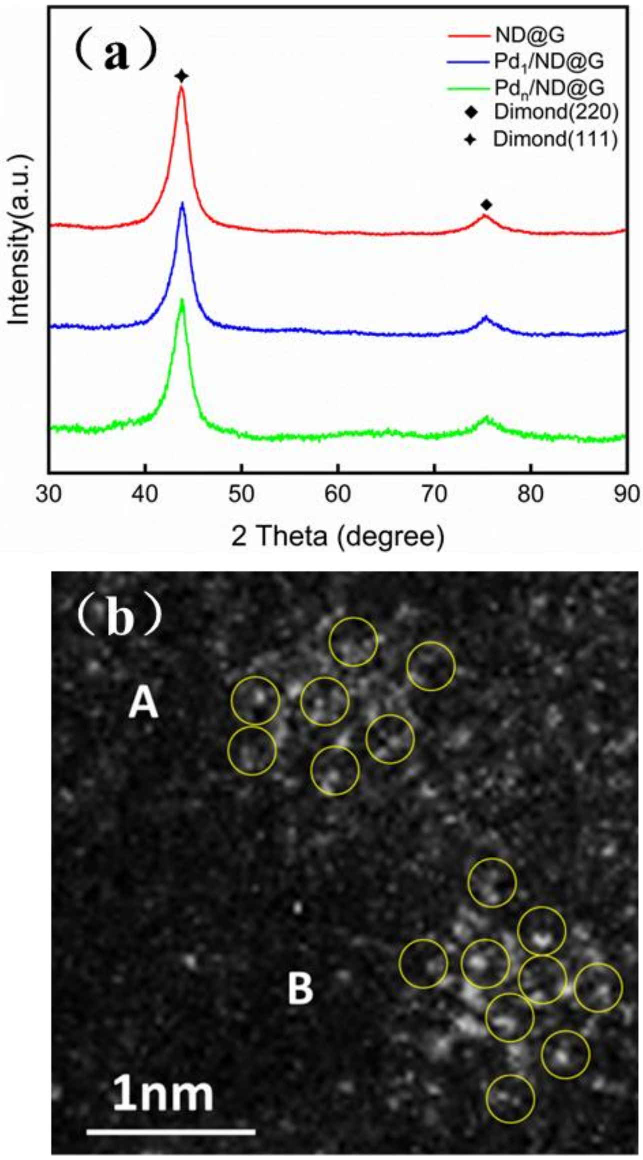

Supplementary Figure 2. (a) XRD profiles of Pd_1/ND@G, Pd_n/ND@G and pure ND@G support. **(**b) The magnified AC-HAADF-STEM images of two clusters in Figure 1d, the separated small Pd clusters were highlighted by yellow circles.

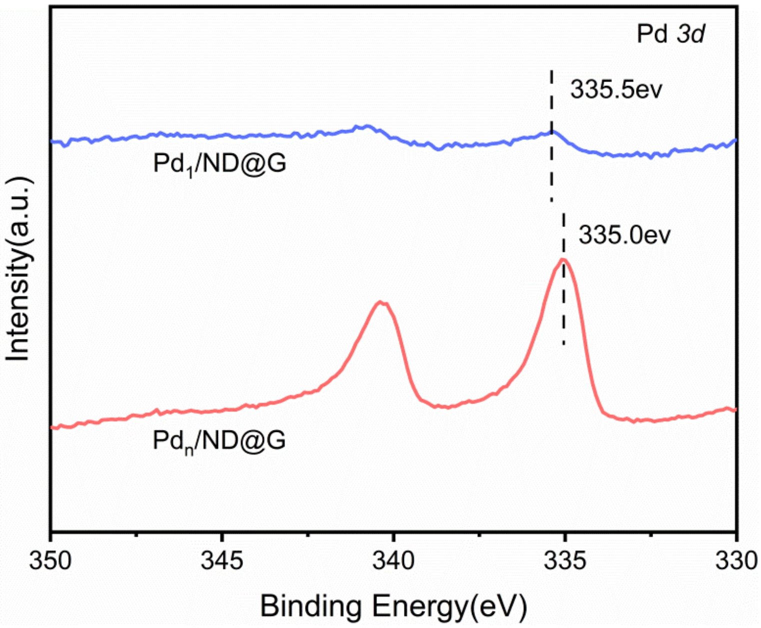

Supplementary Figure 3. XPS spectra of Pd_1/ND@G and Pd_n/ND@G.

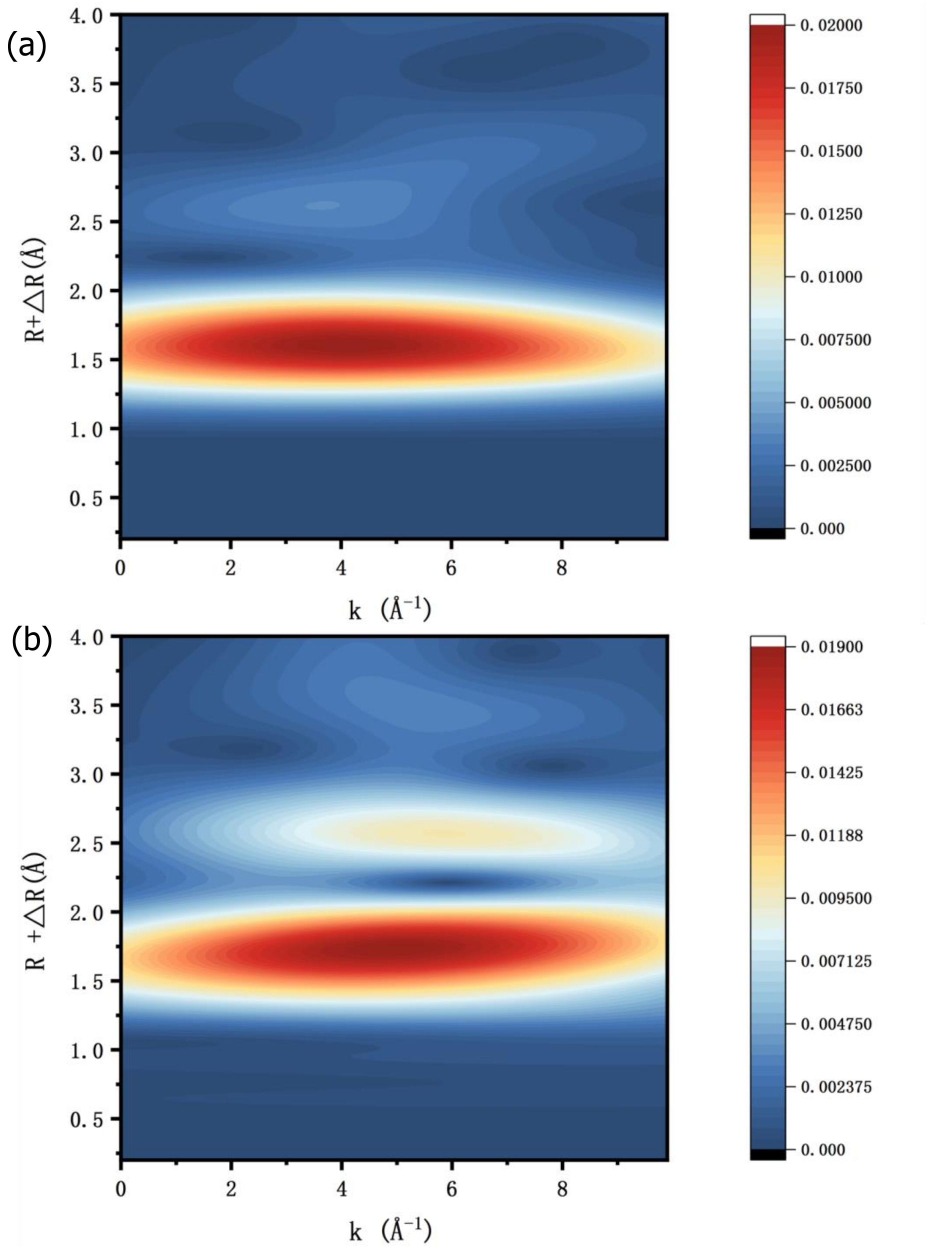

Supplementary Figure 4. (a) WT analysis of Pd_1/ND@G. (b) WT analysis of Pd_n/ND@G.

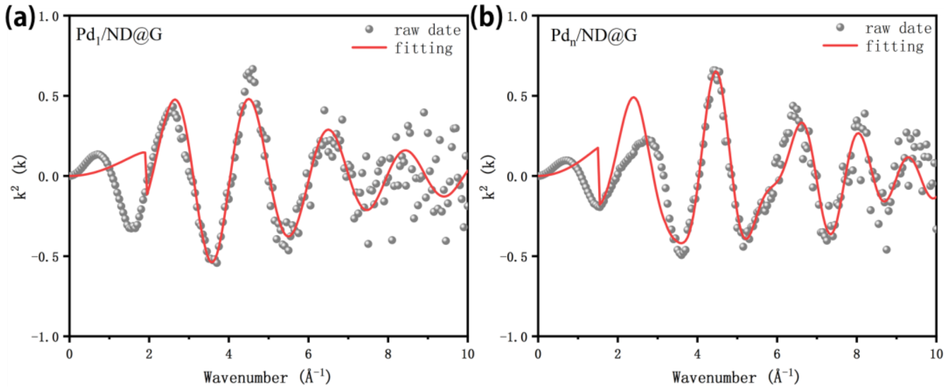

Supplementary Figure 5. Pd K-edge EXAFS fitting results for (a) Pd_1/ND@G and (b) Pd_n/ND@G .

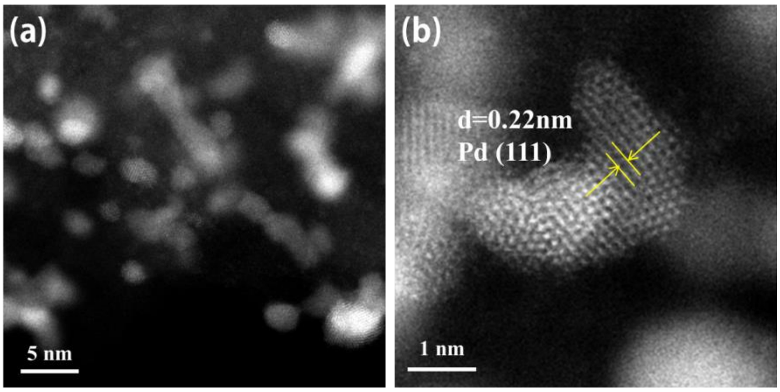

Supplementary Figure 6. (a, b) AC-HAADF-STEM images of the commercial Pd/C catalyst with 5 wt%.

Supplementary Table 2. Structural parameters extracted from quantitative EXAFS curve-fitting (S_0^2= 0.8 – 0.9).

Sample	Shell	C.N.[a]	R(Å)[b]	$\Delta\sigma^2$ (/10^{-3} Å^2) [c]	ΔE_0 (eV) [d]	R factor
Pd foil	Pd-Pd	12.0	2.74	5.86	-6.49	0.001
PdO	Pd-O	4.0	2.01	2.13	-2.62	0.001
Pd_1/ND@G	Pd-Pd	0	-	-	-	0.027
	Pd-C/O	2.6	2.04	1.80	4.660	
Pd_n/ND@G	Pd-Pd	1.9	2.75	5.76	8.603	0.030
	Pd-C/O	2.5	2.02	5.19		

[a] C.N. is the coordination number. [b] R is interatomic distance (the bond length between Pd central atoms and surrounding coordination atoms). [c] σ^2 is Debye-Waller factor (a measure of thermal and static disorder in absorber scatterer distances).[d] ΔE_0 is edge energy shift (the difference between the zero kinetic energy value of the sample and that of the theoretical model.

Supplementary Table 3. Reaction conditions optimization of Pd_1/ND@G for selective catalytic transfer hydrogenation of benzonitriles.

Entry	Catalyst (mg)	T (^oC)	AB. (mmol)	Conversion (%)	Selectivity (%)		
					BA	DBA	TOL
1	10	60	4	51	0	68	0
2	20	60	4	90	0	82	0
3	30	60	4	>99	0	>98	0
4	30	50	4	93	0	84	0
5	30	40	4	25	0	36	0
6	30	60	3	54	0	79	0
7	30	60	2	3	0	0	0

Reaction condition: 0.5 mmol Nitriles;10 ml Methanol; 8 h. Toluene (TOL).

Supplementary Table 4. Catalytic performance of the Pd_1/ND@G catalyst in BN hydrogenation with different solvents.

Entry	Solvent	Conversion (%)	Selectivity (%)		
			BA	DBA	TOL
1	EtOH	48	77	23	0
2	MeOH	>99	0	>98	0
3	i-PrOH	4	0	0	0
4	Hexane	5	0	0	0
5	THF	6	0	0	0

Reaction condition:0.5 mmol Nitriles; 8 h; 30 mg Pd_1/ND@G catalyst; 4 mmol ammonia borane; 60 ^{o}C;10ml solvent.

Supplementary Table 5. Reaction conditions optimization of Pd_n/ND@G for selective catalytic transfer hydrogenation of benzonitriles.

Entry	Catalyst (mg)	T (^oC)	AB. (mmol)	Conversion (%)	Selectivity (%)		
					BA	DBA	TOL
1	5	40	3	80	90	10	0
2	10	40	3	>99	>98	trace	0
3	20	40	3	>99	>98	trace	0
4	10	40	2	78	>98	trace	0
5	10	40	4	>99	>98	trace	0
6	10	30	3	95	>98	0	0
7	10	50	3	93	97	3	0
8	10	60	3	98	96	0	4

Reaction condition: 0.5 mmol Nitriles,10 ml Methanol; 0.5 h.

Supplementary Table 6. Catalytic performance of the Pd_nND@G catalyst in BN hydrogenation with different solvents.

Entry	Solvent	Conversion (%)	Selectivity (%)		
			BA	DBA	TOL
1	EtOH	73	79	trace	15
2	MeOH	>99	>98	trace	0
3	i-PrOH	12	0	0	0
4	Hexane	11	0	0	0
5	THF	10	0	0	0

Reaction condition:0.5 mmol Nitriles; 0.5 h; 10mg Pd_n/ND@G catalyst; 3 mmol ammonia borane; 40 ^oC; 10ml solvent.

Supplementary Table 7. Catalytic performance of different noble metal catalysts in the hydrogenation and transfer hydrogenation of benzonitriles.

Entry	Reaction conditions	Conversion (%)	Selectivity (%)		TOF (h^{-1})	Reference
			BA	DBA		
Pd/Al_2O_3	70 ^oC,1.5 MPa H_2, WHSV = 0.5 h^{-1}	96.7	88.9	0	100	1
Pd/C	60 ^oC, 4 MPa H_2, 0.33 h	50	31	0	218	2
Pd/C	60 ^oC, 0.6 MPa H_2, 2.3 h	100	80	0	180	3
Pd/TiO_2	30 ^oC,0.6 MPa H_2,12.5 h	75	94	0	13	
Pd/η-Al_2O_3	90 ^oC, 1.5 MPa H_2, 4 h	100	91	6	41	4
5Pd-Ni/SiO_2	80 ^oC,0.6 MPa H_2,3 h	100	3.5	96.5	515	5
**Pd_n/ND@G**	**40 ^oC,3mmol AB.(0.1MPa),0.5 h**	**>99**	**>98**	**trace**	**543**	**This work**
**Pd_1/ND@G**	**60 ^oC,4mmol AB.(0.1MPa),8 h**	**>99**	**0**	**>98**	**709**	
Pt /C	105 ^oC,5.5 MPa H_2,2.5 h	100	0	97	780	6
Pt/Al_2O_3	100 ^oC, 1.5 MPa H_2, 24 h	100	12	0	20	7
Pt/C	r.t., 0.34 MPa H_2,4 h	100	0	93	50	8
Rh/C	50 ^oC,2 MPa H_2,10 MPa CO_2,4 h	28.8	25	75	14.4	9
Rh/Al_2O_3		21	22.4	77.6	10	
Ru-NPs	290 ^oC, 6.2 MPa H_2,72 h	100	56.6	0	140	10
Ru/K-Al_2O_3	70 ^oC,0.1 MPa H_2, 82 h	100	93	0	1.2	11
Ir/Al_2O_3	100 ^oC,2.1 MPa H_2	20	36	52	66	12

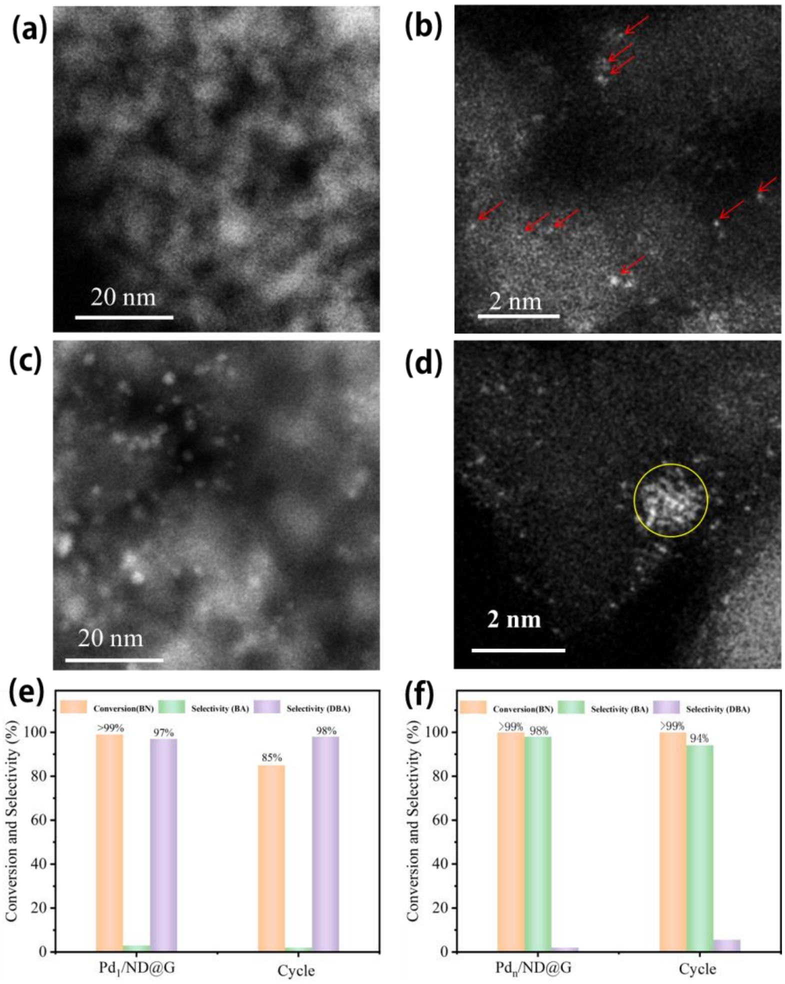

Supplementary Figure 7. The AC-HAADF-STEM images of the Pd_1/ND@G catalyst at low (a) and high (b) magnification after the transfer hydrogenation of benzonitrile. The AC-HAADF-STEM images of the Pd_n/ND@G catalyst at low (c) and high (d) magnification after the transfer hydrogenation of benzonitrile. (e) The recycling experiments of the Pd_1/ND@G catalyst used for the transfer hydrogenation of benzonitrile. Reaction conditions: solvent, methanol, 10 mL; BN, 0.5 mmol; catalyst, 60 mg; AB, 4 mmol; Temperature, 60 ^{o}C; Time, 8 h. (f) The recycling experiments of the $Pd_{n/}$ND@G catalyst used for the transfer hydrogenation of benzonitrile. Reaction conditions: solvent, methanol, 10 mL; BN, 0.5 mmol; catalyst, 10 mg; AB, 3mmol; Temperature, 40 ^{o}C; Time, 30 min.

Supplementary Table 8. Relative configurations and energies of different stationary intermediates on Pd_1/ND@G.

Species	Configurations and Energy
PhCN*	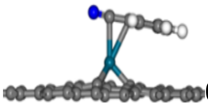 0.50eV 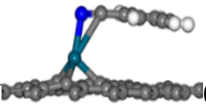 0.15eV 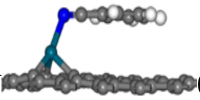 0.00eV
PhCN*+H_2*	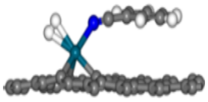 0.50 eV
PhCN*+2H*	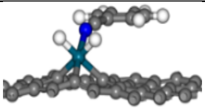 0.47 eV 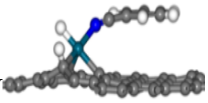 0.00 eV
PhCNH*+H*	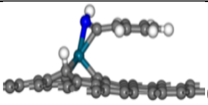 0.26eV 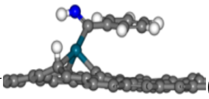 0.00eV 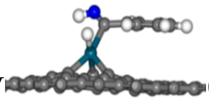 0.35eV 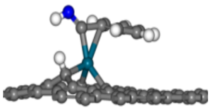 0.34 eV
PhCHNH*	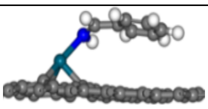 0.26 eV 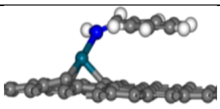 0.00 eV
PhCHNH*+H_2*	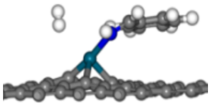
PhCHNH*+2H*	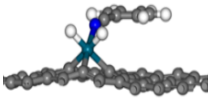 0.47 eV 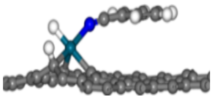 0.00 eV
$PhCHNH_2$*+H*	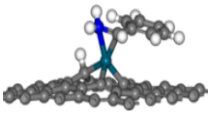 0.00 eV 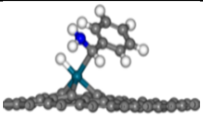 0.67 eV
$PhCH_2NH_2$*	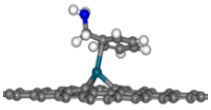

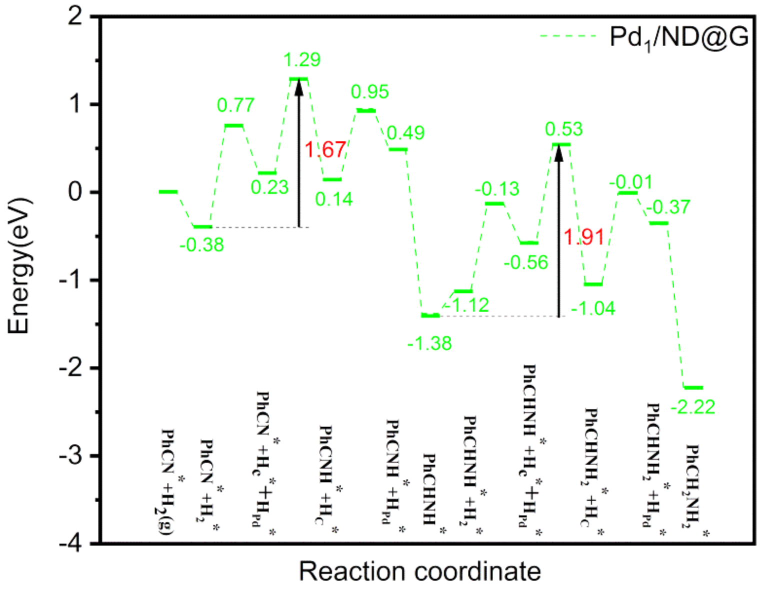

Supplementary Figure 8. The energies of species in the process of hydrogenation reaction from benzonitrile to benzylamine on Pd_1/ND@G.

Supplementary Table 9. Relative configurations and energies of different stationary intermediates on Pd_n/ND@G.

Species	Configurations and Energy
PhCN*	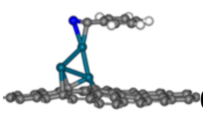 0.76 eV 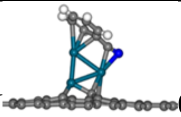 0.41 eV 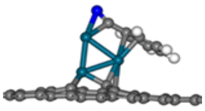 0.18 eV 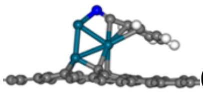 0.00 eV 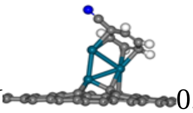 0.20 eV
PhCN*+2H	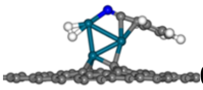 0.00 eV 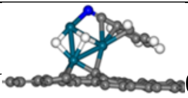 0.10 eV 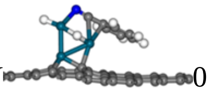 0.96 eV 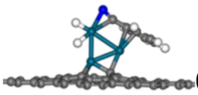 0.34 eV
PhCNH*+H*	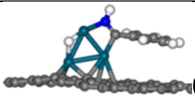 0.00 eV 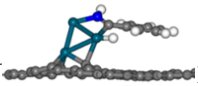 1.13 eV
PhCNH*+H*	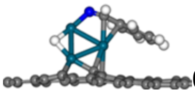 0.94 eV 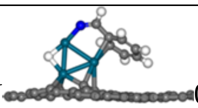 0.39 eV
PhCHNH*	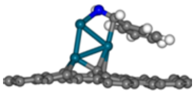
PhCHNH*+2H	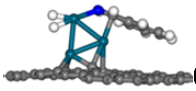 0.00 eV 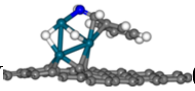 0.30 eV
$PhCHNH_2$*+H*	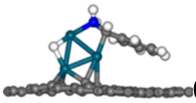 0.00 eV 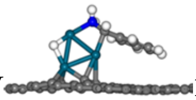 1.03 eV
$PhCH_2NH$*+H*	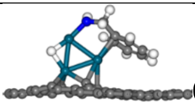 0.40 eV
$PhCH_2NH_2$*	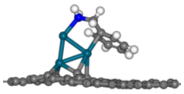

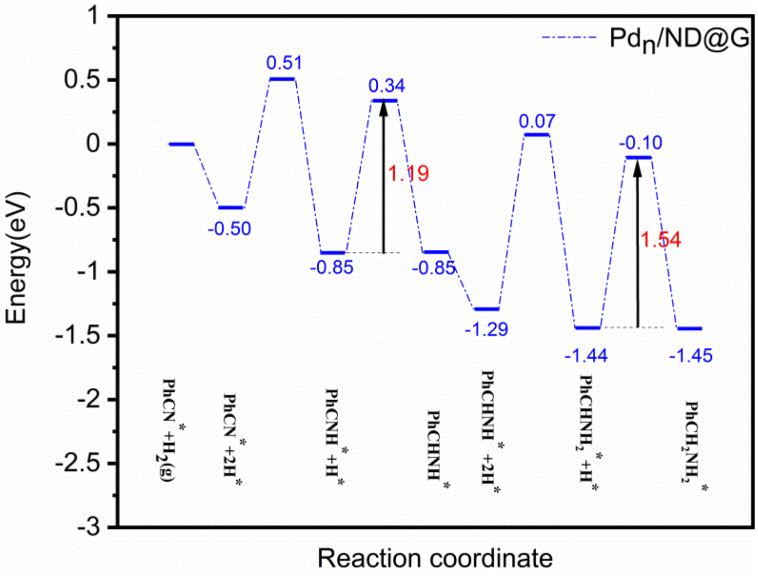

Supplementary Figure 9. The energies of species in the process of hydrogenation reaction from benzonitrile to benzylamine on Pd_n/ND@G.

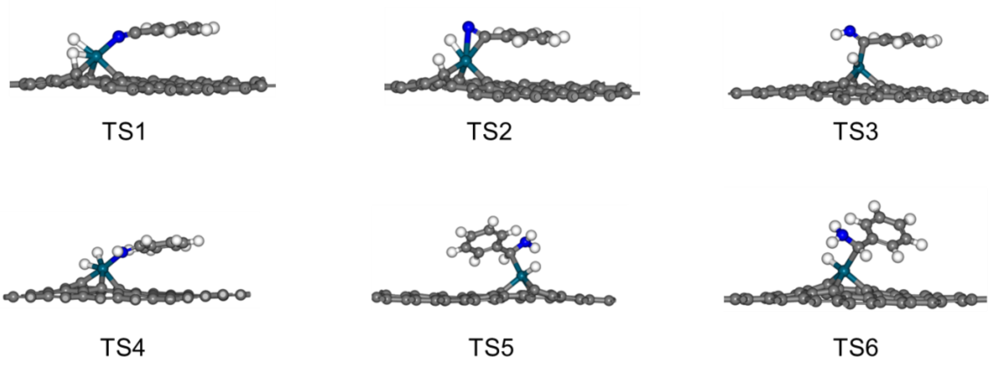

Supplementary Figure 10. Structures of key transition states of benzonitrile hydrogenation on Pd_1/ND@G.

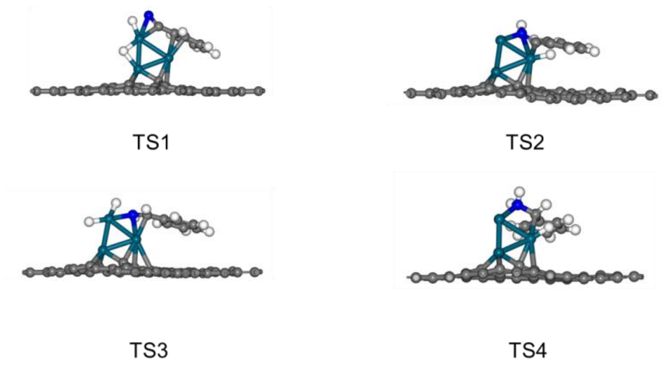

Supplementary Figure 11. Structures of key transition states of benzonitrile hydrogenation on Pd_n/ND@G.

Supplementary Table 10. Step by step barrier (Ea, eV) and enthalpy energy change (ΔE, eV) of benzonitrile to benzylamine on Pd_1-Gr.

Number	Elementary reactions	E_a (eV)	ΔE (eV)
1	PhCN= PhCN*	-0.77	-
2	H_2(g) + PhCN* = PhCN*+H_2*	-0.77	-
3	PhCN*+H_2* = H*(C)+H*(Pd)+PhCN*	0.61	1.15
4	H*(C)+H*(Pd)+PhCN* = H(C)+PhCNH*	-0.09	1.06
5	H*(C)+PhCNH*= H*(Pd)+PhCNH*	0.35	0.81
6	H*(Pd)+PhCNH*= PhCHNH*	-1.87	-
7	H_2(g) + PhCHNH= H_2*+PhCHNH*	-0.13	-
8	H_2*+PhCHNH* = H*(C)+H*(Pd)+PhCHNH*	0.56	0.99
9	H*(C)+H*(Pd)+PhCHNH* = H*(C)+PhCHNH_2*	-0.48	1.09
10	H*(C)+PhCHNH_2*= H*(Pd)+PhCHNH_2*,	0.67	1.03
11	H*(Pd)+PhCHNH_2* = PhCH_2NH_2*	-1.06	-

Supplementary Table 11. Step by step barrier (Ea, eV) and enthalpy energy change (ΔE, eV) of benzonitrile to benzylamine on Pd_n-Gr.

Number	Elementary reactions	E_a (eV)	ΔE (eV)
1	PhCN= PhCN*	-1.58	-
2	H_2(g) + PhCN* = PhCN*+2H*	-0.89	-
3	PhCN*+2H* = PhCNH*+H*	-0.35	1.01
4	PhCNH*+H* = PhCHNH*	0.00	1.19
5	H_2(g) + PhCHNH* = 2H* + PhCHNH*	-0.83	-
6	2H* + PhCHNH* = $PhCHNH_2$*+H*	-0.15	1.36
7	$PhCHNH_2$*+H* = $PhCH_2NH_2$*	-0.01	1.34

Characterization data of products

Primary amines

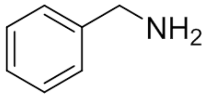

Benzylamine (yellowish liquid)[13]

Isolated yield: 93%

^{1}H NMR (600 MHz, Chloroform-d) δ 7.33-7.26 (m, 5H), 3.83 (s, 2H), 2.03 (s, active H).

^{13}C NMR (151 MHz, Chloroform-d) δ 128.97, 128.77, 128.15, 127.72, 45.34.

GC-MS: *m/z* (%) 107 (60) $[M]^+$, 106 (100), 91 (15), 79 (48), 77 (25), 65 (10), 51 (18).

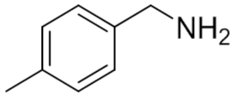

p-tolylmethanamine (colorless liquid)[14]

Isolated yield: 89%

^{1}H NMR (600 MHz, Chloroform-d) δ 7.19 (d, J = 7.9 Hz, 2H), 7.17 (d, J = 7.8 Hz, 2H), 3.82 (s, 2H), 2.34 (s, 3H), 1.74 (s, active H).

^{13}C NMR (151 MHz, Chloroform-d) δ 140.17, 136.60, 129.37, 127.23, 46.30, 21.20.

GC-MS: *m/z* (%) 121 (23) $[M]^+$, 120 (62), 104 (100), 91 (65), 77 (42), 65 (28), 51 (22).

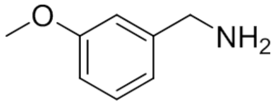

(3-methoxyphenyl)methanamine (yellowish liquid)[14]

Isolated yield: 91%

^{1}H NMR (600 MHz, Chloroform-d) δ 7.28 – 7.20 (m, 1H), 6.86 (d, J = 14.4 Hz, 2H), 6.78 (d, J = 2.2 Hz, 1H), 3.81 (s, 2H), 3.79 (s, 3H), 1.76 (s, 2H).

^{13}C NMR (151 MHz, Chloroform-d) δ 159.81, 144.87, 129.52, 119.31, 112.59, 112.20, 55.14, 46.38.

GC-MS: *m/z* (%) 137 (65) $[M]^+$, 136 (100), 121 (24), 106 (64), 94 (42), 77 (56), 65 (32), 51 (30).

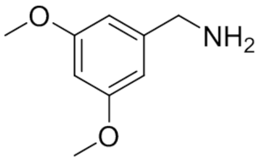

(3,5-dimethoxyphenyl)methanamine (yellowish liquid) (CAS: 1391721-68-8)

Isolated yield: 90%

^{1}H NMR (600 MHz, Chloroform-d) δ 6.45 (s, 2H), 6.32 (s, 1H),3.76（s，2H）3.75 (s, 6H), 1.94 (s, 2H).

^{13}C NMR (151 MHz, Chloroform-d) δ 160.98, 145.60, 104.91, 98.73, 55.23, 46.50.

GC-MS: *m/z* (%) 167 (53) $[M]^+$, 166 (65), 146 (63), 136 (30), 105 (100), 77 (50), 65 (27), 51 (25).

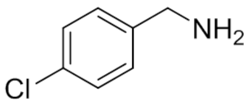

(4-chlorophenyl)methanamine (colorless liquid)[15]

Isolated yield: 83%

^{1}H NMR (600 MHz, Chloroform-d) δ 7.30 (d, J = 9.2 Hz, 2H), 7.25 (d, J = 14.5 Hz, 2H), 3.84 (s, 2H), 1.73 (s, active H).

^{13}C NMR (151 MHz, Chloroform-d) δ 141.61, 132.64, 128.75, 128.61, 45.87.

GC-MS: *m/z* (%) 141 (8) $[M]^+$, 140 (35), 106 (100), 89 (15), 77 (45), 63 (12), 51 (25).

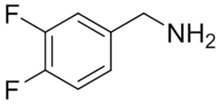

(3,4-difluorophenyl)methanamine (colorless liquid) (CAS: 72235-53-1)

Isolated yield: 81%

^{1}H NMR (600 MHz, Chloroform-d) δ 7.13 (d, J = 2.8 Hz, 1H), 7.05 (d, J = 10.2 Hz, 1H), 7.03 (s, 1H),3.84 (s, 2H)，1.72 (s, active H).

^{13}C NMR (151 MHz, Chloroform-d) δ 150.19 (d, J = 12.8 Hz), 149.65 (d, J = 12.8 Hz), 140.20, 123.21–122.77 (m), 117.25 (d, J = 16.8 Hz), 116.07 (d, J = 17.2 Hz), 45.55.

GC-MS: *m/z* (%) 143 (15) $[M]^+$, 142 (60), 123 (100), 95 (17), 75 (22), 63 (45), 51 (10).

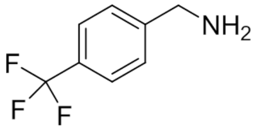

(4-(trifluoromethyl)phenyl)methanamine (brown liquid)[13]

Isolated yield: 94%

^{1}H NMR (600 MHz, Chloroform-d) δ 7.59 (d, J = 8.0 Hz, 2H), 7.44 (d, J = 8.0 Hz, 2H), 3.94 (s, 2H), 1.76 (s, 2H).

^{13}C NMR (151 MHz, Chloroform-d) δ 147.04, 128.65, 127.47, 125.80, 124.69 (m), 46.06.

GC-MS: *m/z* (%) 175 (46) [M]$^+$, 174 (87), 156 (20), 127 (59), 106 (100), 77 (26), 69 (27), 51 (26).

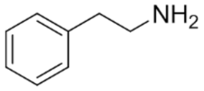

Phenethylamine (yellowish liquid)[13]

Isolated yield: 85%

^{1}H NMR (600 MHz, Chloroform-d) δ 7.32 (d, J = 12.3 Hz, 2H), 7.28 (s, 2H), 7.21 (s, 1H), 3.52 (dd, J = 14.5, 8.3 Hz, 1H), 2.82 (d, J = 6.2 Hz, 2H), 1.94 (s, 2H).

^{13}C NMR (151 MHz, Chloroform-d) δ139.01, 128.84 (d, J = 13.0 Hz), 126.67,40.78，35.76.

GC-MS: *m/z* (%) 121 (21) [M]$^+$, 120 (30), 91 (100), 77 (21), 65 (68), 51 (26).

Secondary amines

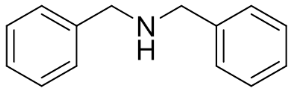

Dibenzylamine (colorless liquid)[16]

Isolated yield: 91%

^{1}H NMR (600 MHz, Chloroform-d) δ 7.29 (d, J = 6.7 Hz, 8H), 7.21 (s, 2H), 3.76 (s, 4H), 1.92 (s, 1H).

^{13}C NMR (151 MHz, Chloroform-d) δ 140.23, 128.42, 128.19, 126.98, 53.13.

GC-MS:*m/z* (%) 197 (12) [M]$^+$, 196 (14), 106 (77), 91 (100), 65 (24), 51 (10).

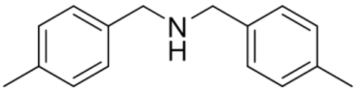

bis(4-methylbenzyl)amine (yellowish liquid)[16]

Isolated yield: 82%

^{1}H NMR (600 MHz, Chloroform-d) δ 7.20 (d, J = 7.9 Hz, 4H), 7.12 (d, J = 7.8 Hz, 4H), 3.73 (s, 4H), 2.32 (s, 6H), 2.11 (s, 1H).

^{13}C NMR (151 MHz, Chloroform-d) δ 137.13, 136.59, 129.16, 128.25, 52.79, 21.18.

GC-MS: *m/z* (%) 225 (8) $[M]^+$, 120 (65), 105 (100), 91 (30), 77 (26), 65 (12), 51 (10).

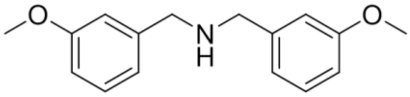

bis(3-methoxybenzyl)amine (yellowish liquid)[17]

Isolated yield: 81%

^{1}H NMR (600 MHz, Chloroform-d) δ 7.22 (t, J = 8.0 Hz, 2H), 6.90 (d, J = 2.1 Hz, 4H), 6.79 (d, J = 8.2 Hz, 2H), 3.78 (s, 6H), 3.76 (s, 4H), 2.16 (s, 1H).

^{13}C NMR (151 MHz, Chloroform-d) δ 159.82, 141.76 129.44, 120.54, 113.70, 112.60, 55.22, 53.01.

GC-MS: *m/z* (%) 257 (1) $[M]^+$, 136 (83), 122 (100), 121 (75), 91 (39), 77 (27), 65 (23), 51 (10).

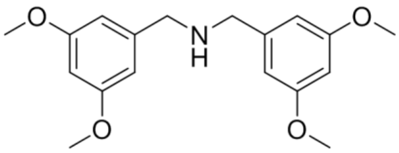

bis(3,5-dimethoxybenzyl)amine (white solid) (CAS:256225-28-2)

Isolated yield: 89%

^{1}H NMR (600 MHz, Chloroform-d) δ 6.50 (s, 4H), 6.36 (s, 2H), 3.77 (s, 12H), 3.73 (s, 4H), 2.12 (s, 1H).

^{13}C NMR (151 MHz, Chloroform-d) δ 160.79, 142.63, 106.97, 98.92, 55.13, 53.04.

GC-MS: *m/z* (%) 317 (1) $[M]^+$, 166 (28), 152 (100), 91 (5), 77 (15), 65 (10), 51 (3).

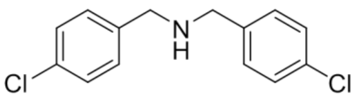

bis(4-chlorobenzyl)amine (colorless liquid)[18]

Isolated yield: 76%

^{1}H NMR (600 MHz, Chloroform-d) δ 7.27 (d, J = 8.4 Hz, 4H), 7.24 (d, J = 8.4 Hz, 4H), 3.72 (s, 4H), 1.90 (s, 1H).

^{13}C NMR (151 MHz, Chloroform-d) δ 138.53, 132.81, 129.56, 128.62, 52.35.

GC-MS: *m/z* (%) 265 (5) $[M]^+$, 140 (42), 125 (100), 89 (13), 77 (15), 63 (10), 51 (10).

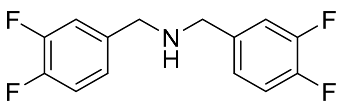

bis(3,4-difluorobenzyl)amine (colorless solid) (CAS: 1284842-52-9)

Isolated yield: 80%

^{1}H NMR (600 MHz, Chloroform-d) δ 7.22 – 7.14 (m, 2H), 7.09 (dt, J = 10.1, 8.2 Hz, 2H), 7.04 (dd, J = 8.0, 4.1 Hz, 2H), 3.74 (s, 4H), 1.82 (s, 1H).

^{13}C NMR (151 MHz, Chloroform-d) δ 151.27 (d, J = 12.5 Hz), 149.63 (d, J = 12.9 Hz), 148.72 (d, J = 12.8 Hz), 137.18, 123.96, 117.03 (dd, J = 32.0, 17.2 Hz), 52.05.

GC-MS: *m/z* (%) 269 (3) $[M]^+$, 136 (100), 121 (24), 106 (64), 94 (42), 77 (56), 65 (32), 51 (30).

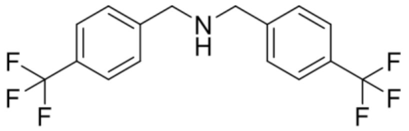

bis(4-(trifluoromethyl)benzyl)amine (colourless liquid)[18]

Isolated yield: 83%

^{1}H NMR (600 MHz, Chloroform-d) δ 7.59 (d, J = 8.2 Hz, 4H), 7.47 (d, J = 8.1 Hz, 4H), 3.86 (s, 4H), 1.95 (s, 1H).

^{13}C NMR (151 MHz, Chloroform-d) δ 144.11, 129.56 (d, J = 35.9 Hz), 128.47, 126.87 (d, J = 18.5 Hz), 125.51, 52.69.

GC-MS: *m/z* (%) 333 (1) $[M]^+$, 142 (32), 127 (100), 101 (15), 77 (5), 63 (8), 51 (2).

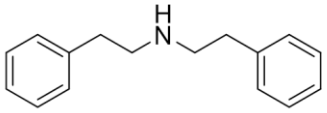

Diphenethylamine (yellowish liquid)[18]

Isolated yield: 89%

^{1}H NMR (600 MHz, Chloroform-d) δ 7.25 (t, J = 7.5 Hz, 4H), 7.22 – 7.17 (m, 2H), 7.15 (d, J = 8.0 Hz, 4H), 2.93 (s, 4H), 2.77 (t, J = 7.1 Hz, 4H), 1.54 (s, 1H).

^{13}C NMR (151 MHz, Chloroform-d) δ 140.01, 128.74, 128.52, 126.20, 50.95, 36.46.

GC-MS: *m/z* (%) 225 (8) $[M]^+$, 134 (95), 105 (100), 77 (27), 65 (16), 51 (14).

Copies of ^{1}H and ^{13}C NMR spectrums：

Primary amines

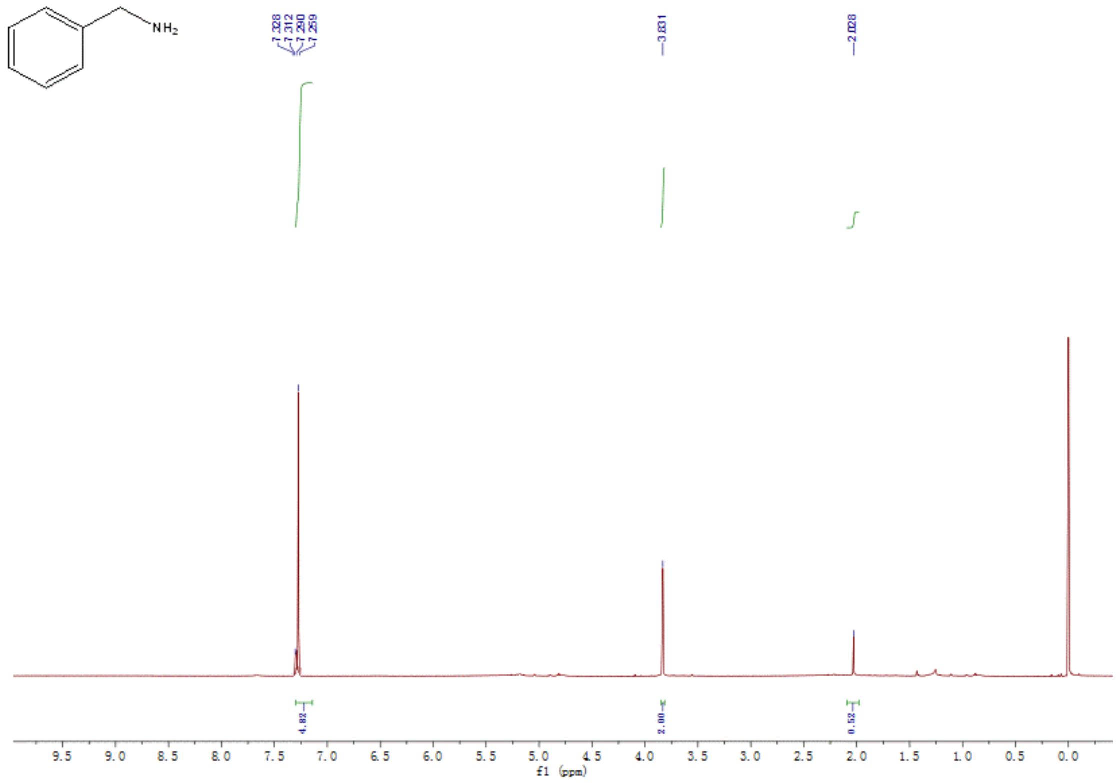

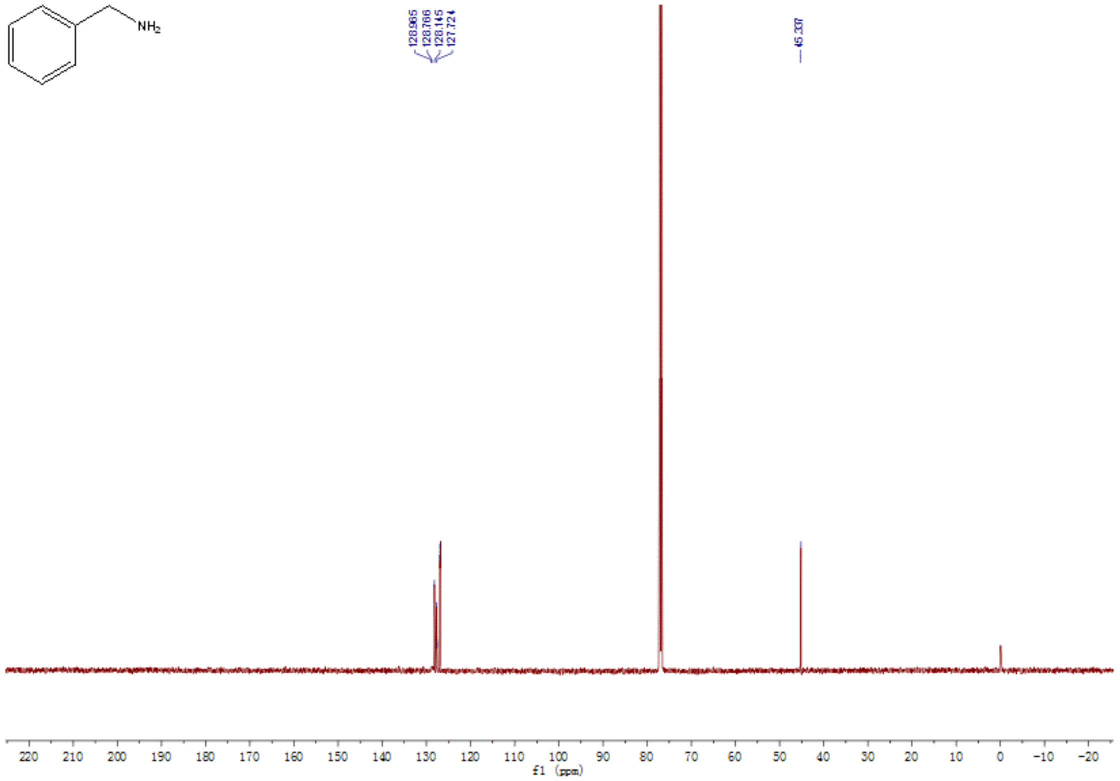

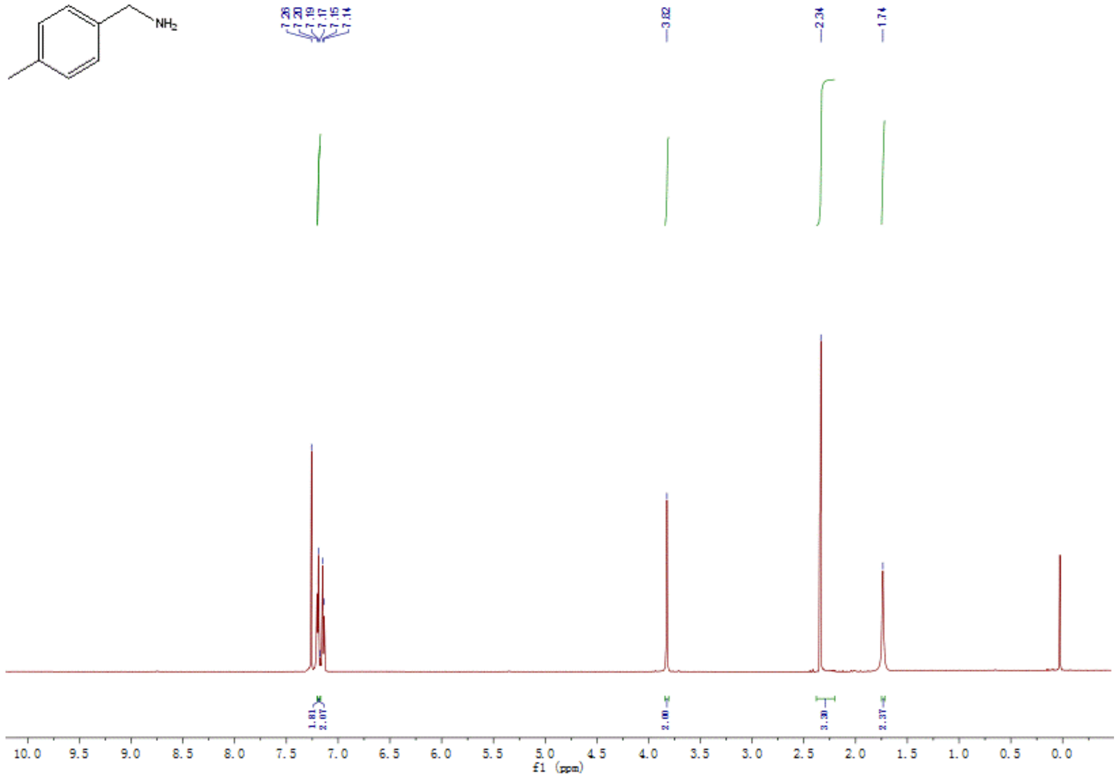
NH2
7.26
7.20
7.19
7.17
7.15
7.14
3.82
2.34
1.74
1.81
2.07
2.00
3.30
2.37
f1 (ppm)

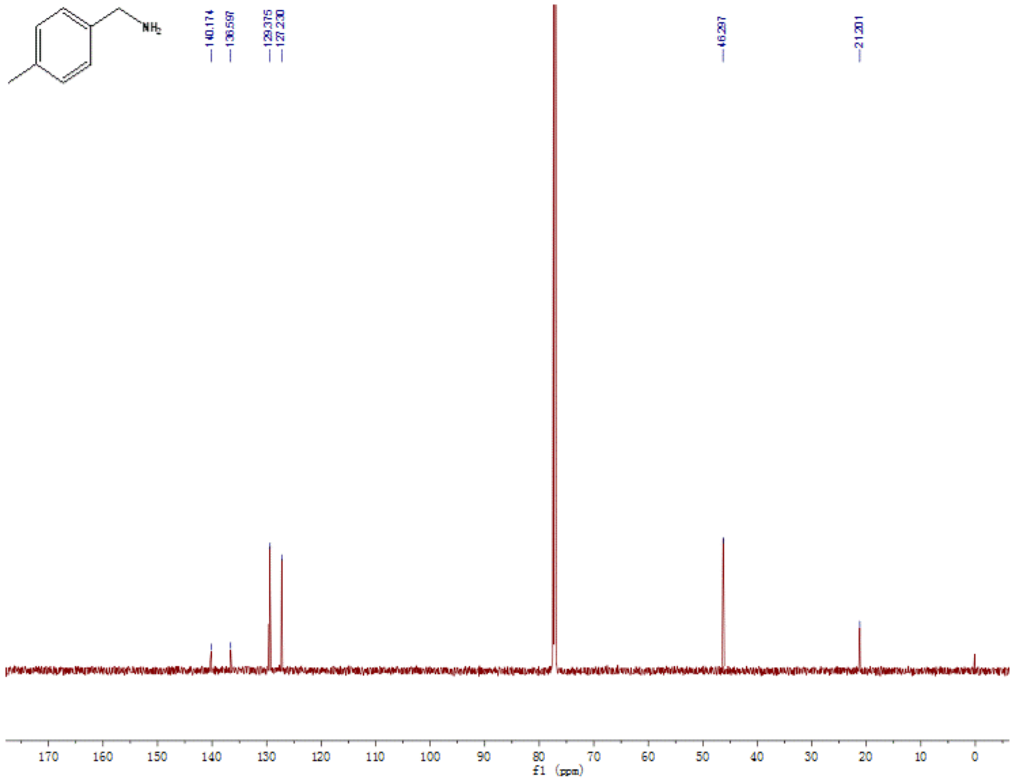
NH2
140.174
136.597
129.375
127.230
46.297
21.201
f1 (ppm)

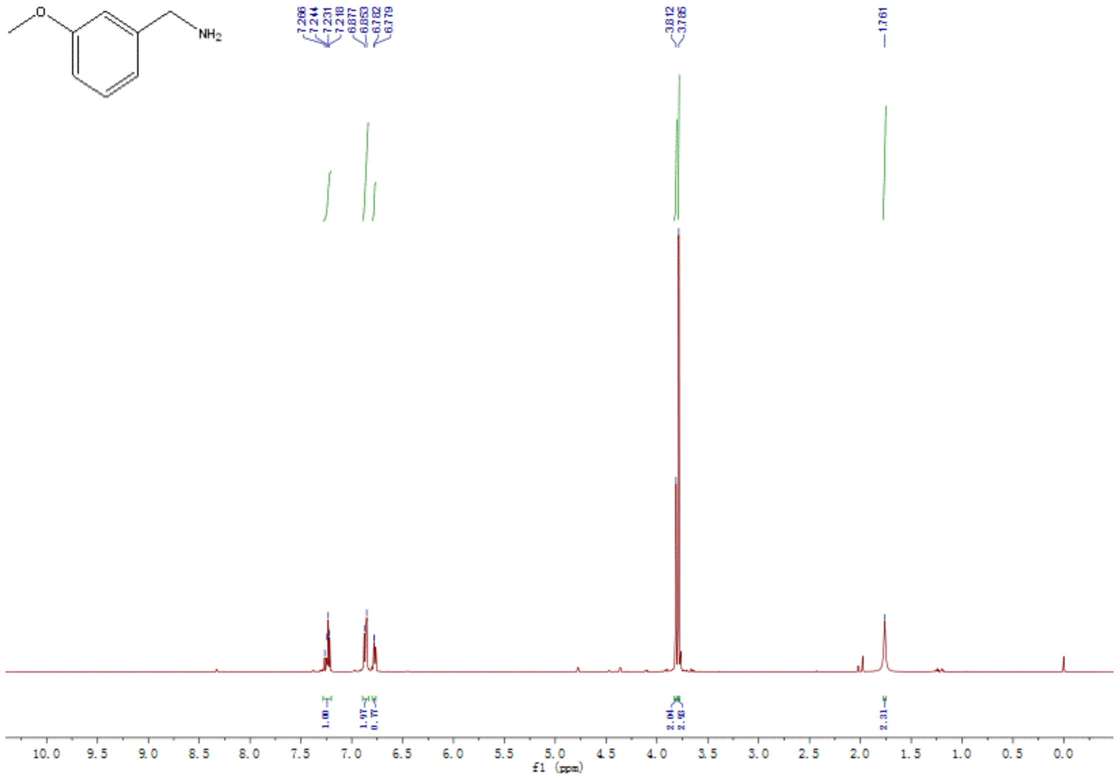
NH2
O
7.266
7.244
7.231
7.218
6.877
6.853
6.782
6.779
3.812
3.795
1.761
1.00
1.97
0.77
2.04
2.98
2.31
f1 (ppm)

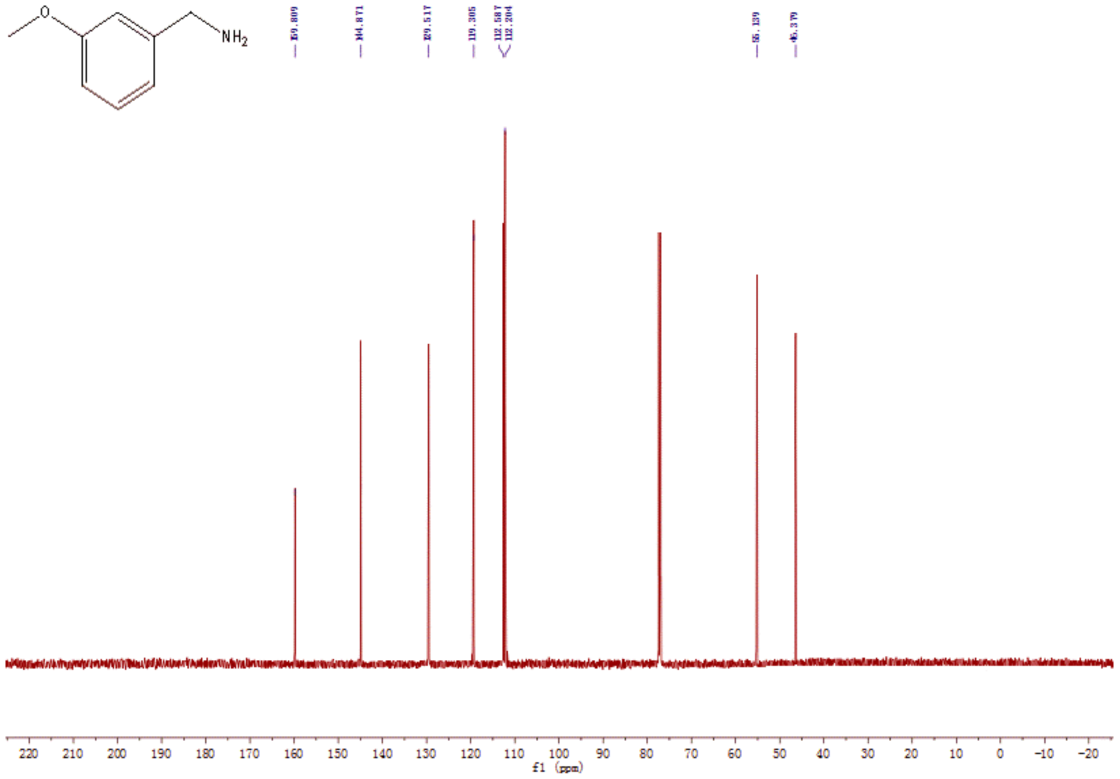
NH2
O
159.809
144.871
129.517
119.305
112.587
112.204
55.139
46.379
f1 (ppm)

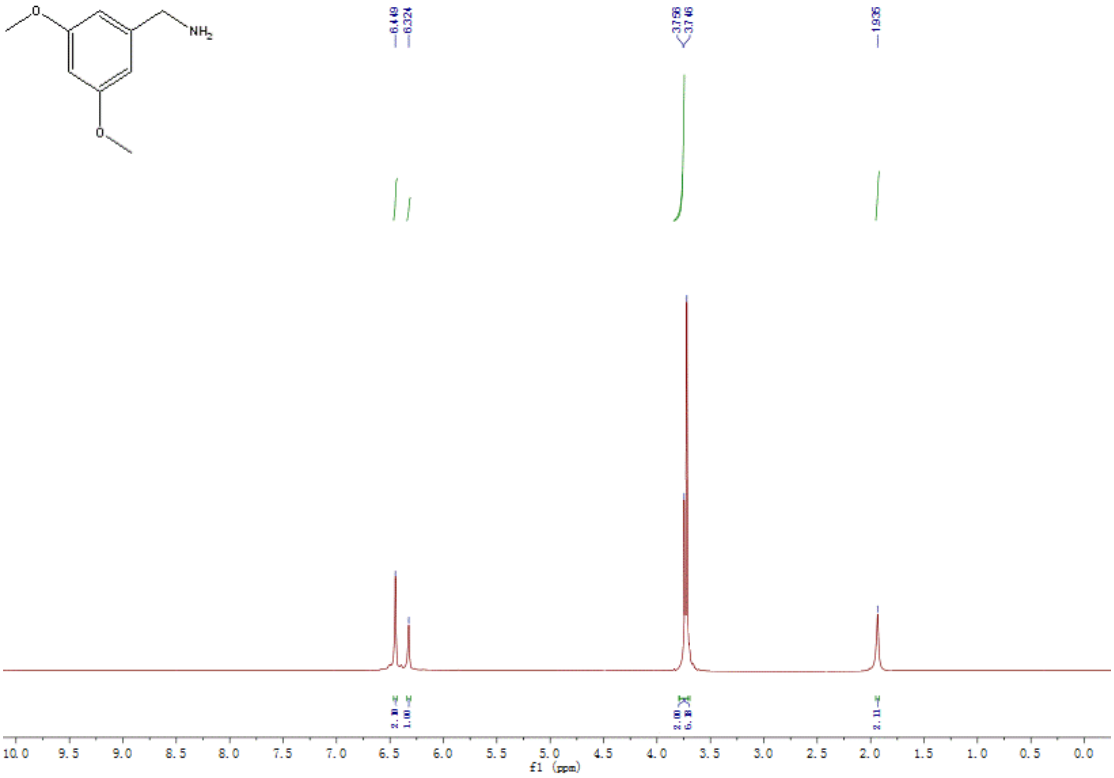
O
O
NH2
6.449
6.324
3.756
3.746
1.935
2.10
1.00
2.00
6.10
2.11
10.0 9.5 9.0 8.5 8.0 7.5 7.0 6.5 6.0 5.5 5.0 4.5 4.0 3.5 3.0 2.5 2.0 1.5 1.0 0.5 0.0
f1 (ppm)

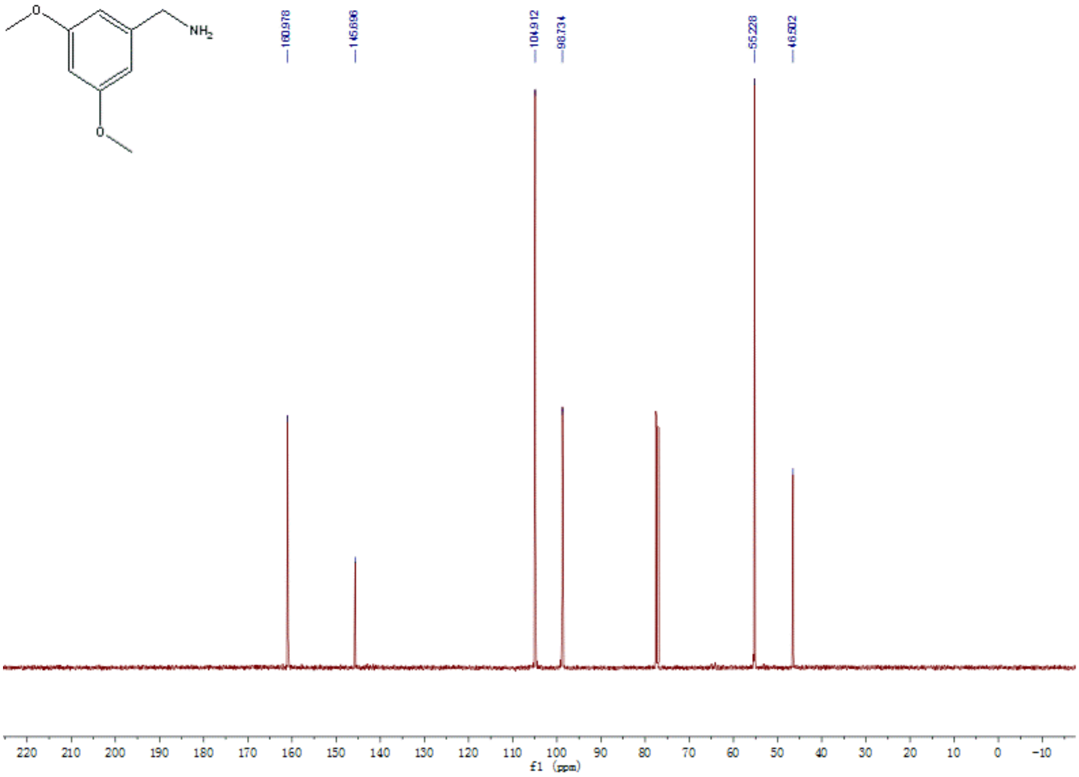
O
O
NH2
160.978
145.696
104.912
98.734
55.228
46.502
220 210 200 190 180 170 160 150 140 130 120 110 100 90 80 70 60 50 40 30 20 10 0 -10
f1 (ppm)

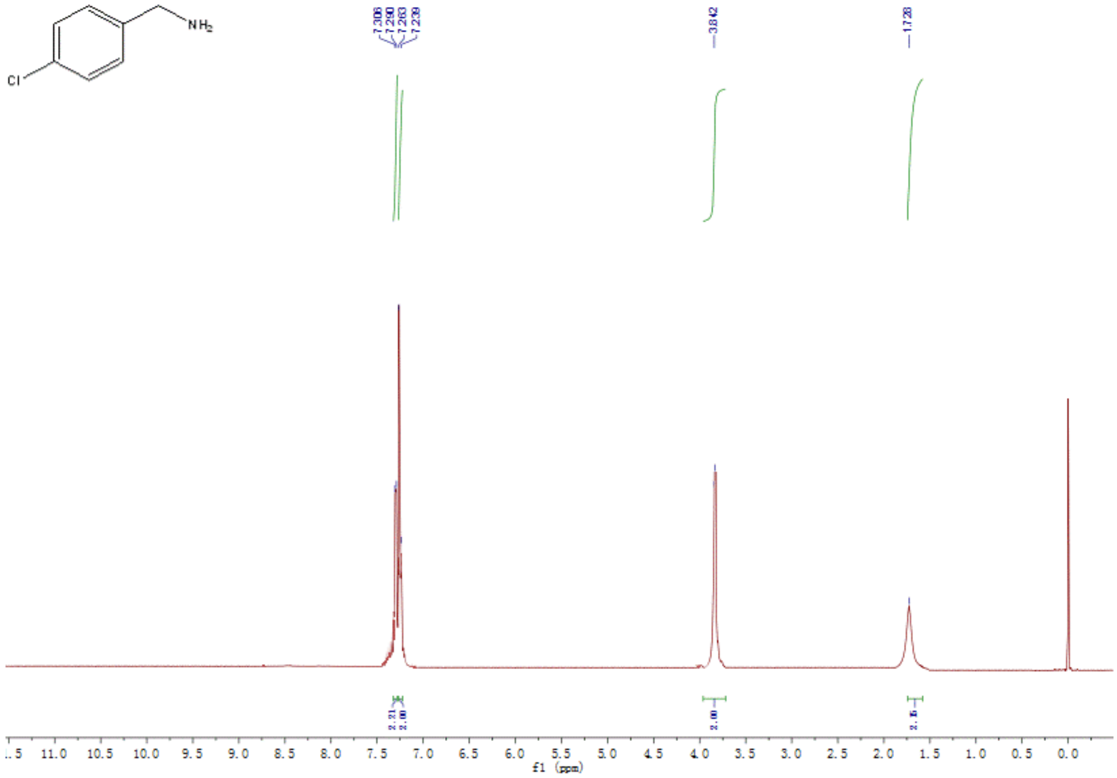
NH2
Cl
7.306
7.290
7.263
7.239
3.842
1.728
2.21
2.00
2.00
2.15
f1 (ppm)

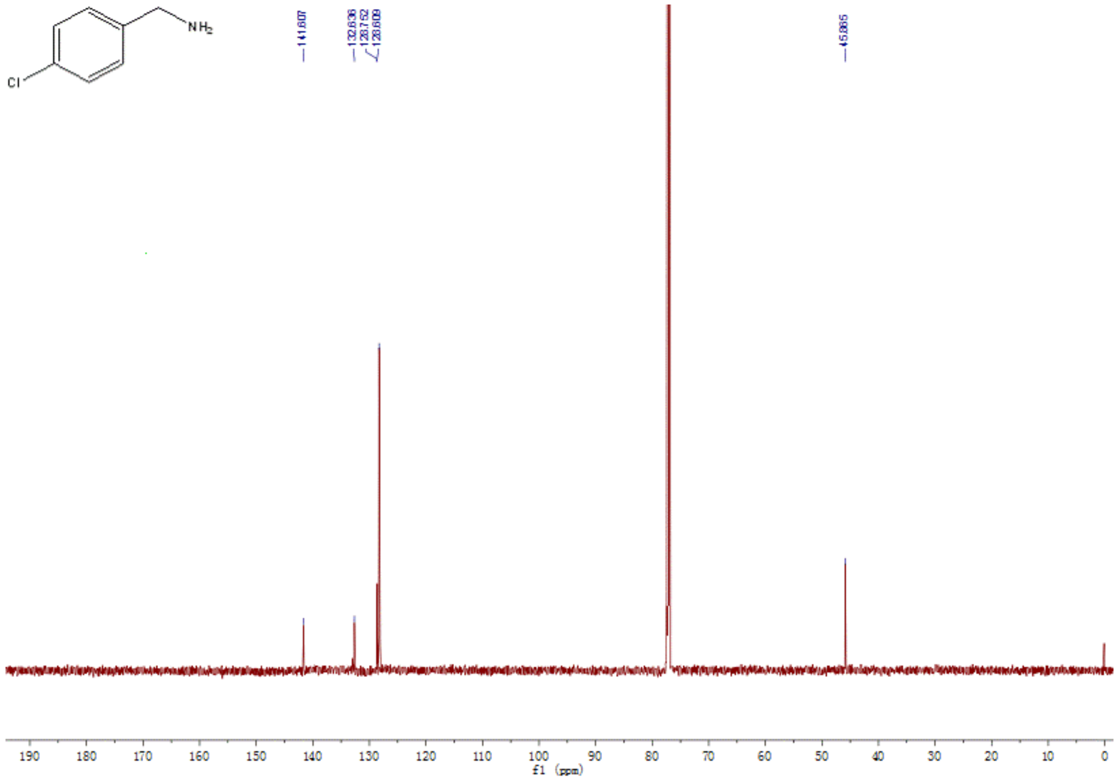
NH2
Cl
141.607
132.636
128.762
128.609
45.865
f1 (ppm)

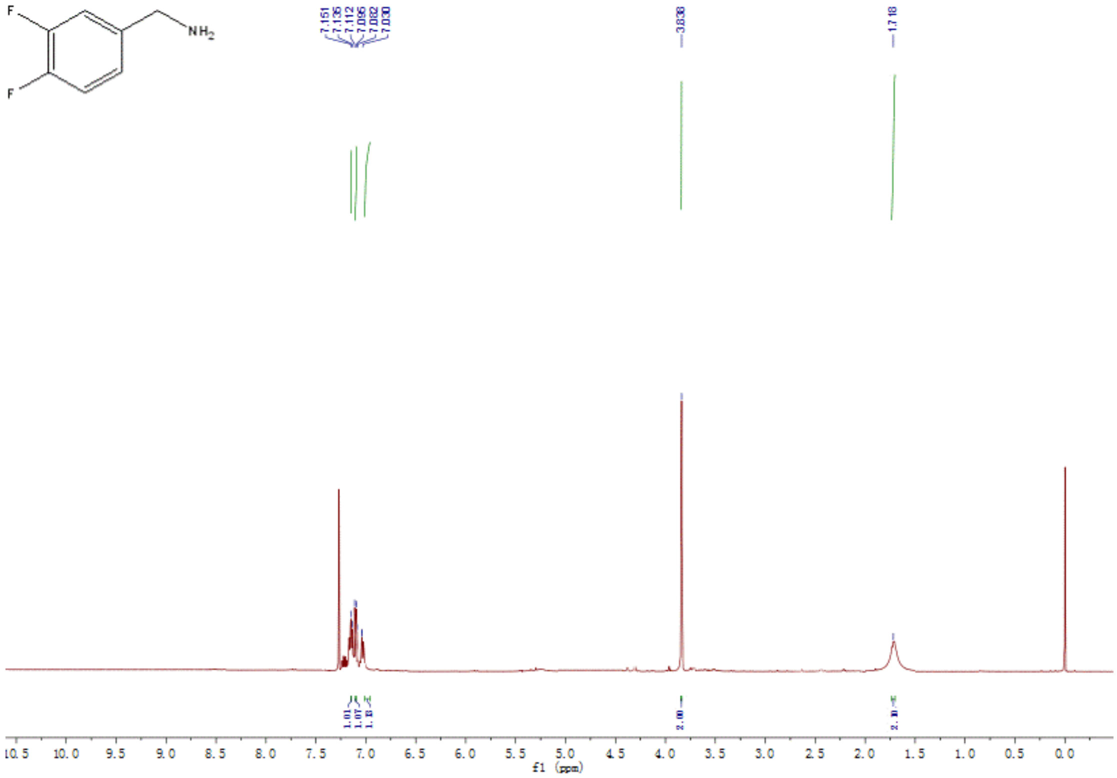
F
F
NH2
f1 (ppm)

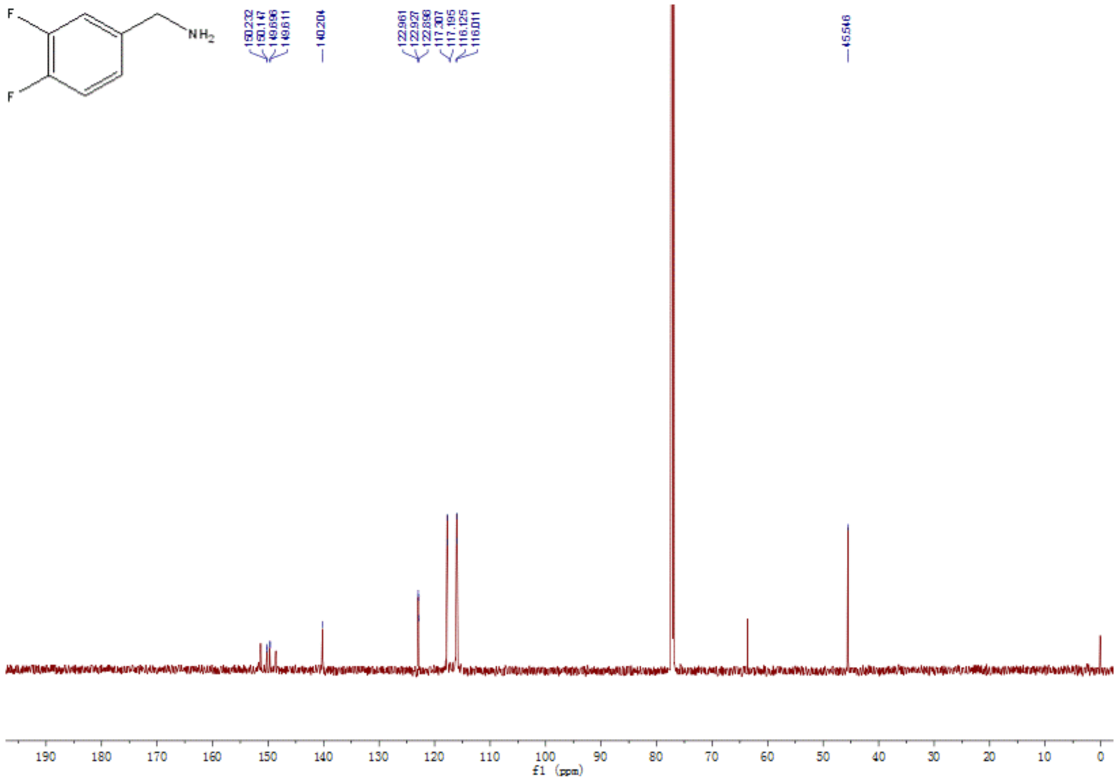
F
F
NH2
f1 (ppm)

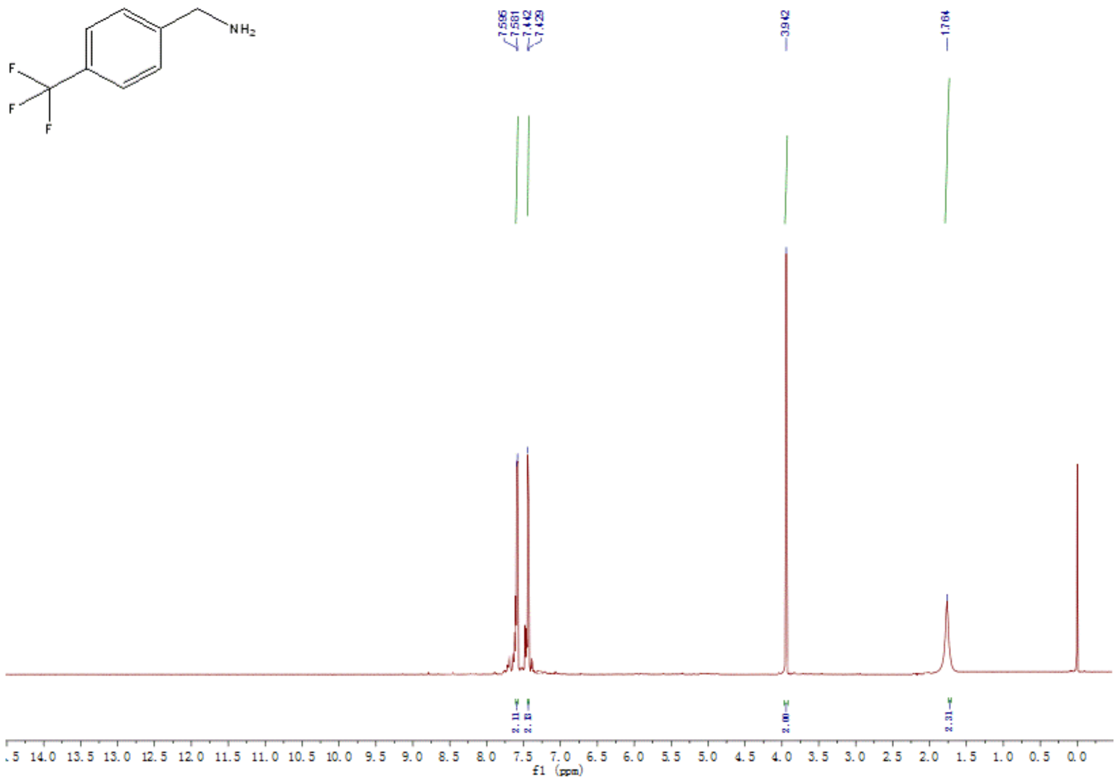
NH2
F
F
F
7.596
7.581
7.442
7.429
3.942
1.764
2.11
2.13
2.00
2.31
f1 (ppm)

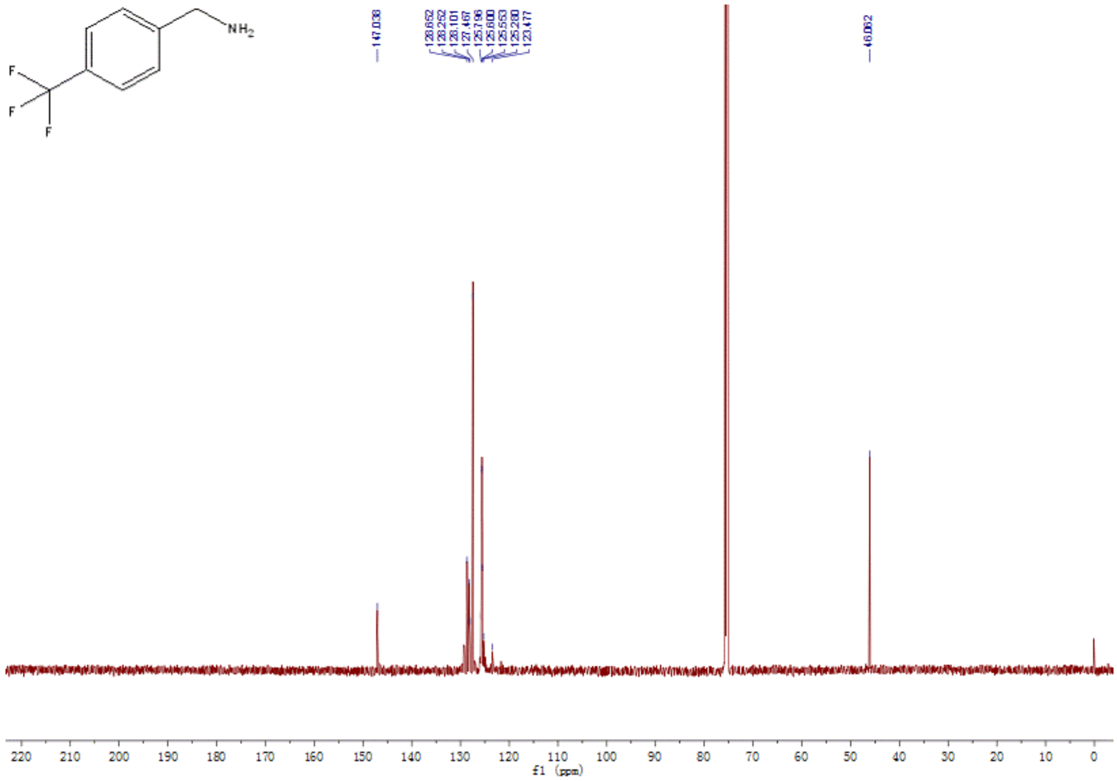
NH2
F
F
F
147.038
128.652
128.252
128.101
127.467
125.796
125.600
125.563
125.280
123.477
46.062
f1 (ppm)

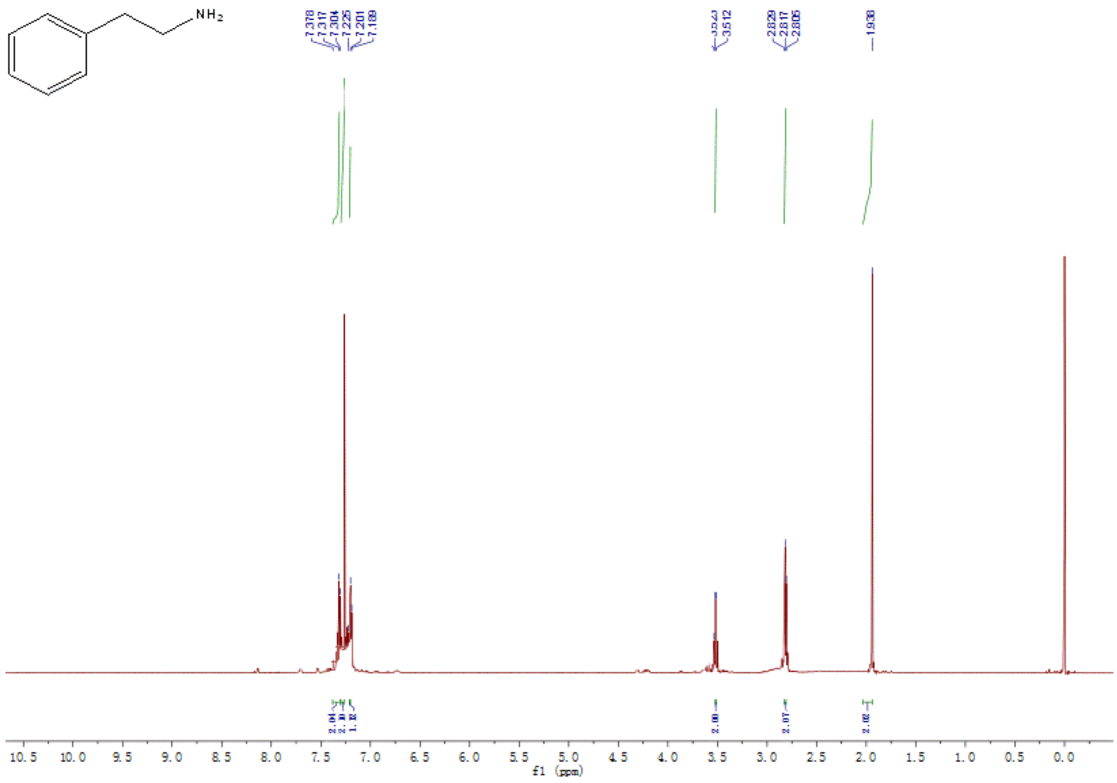

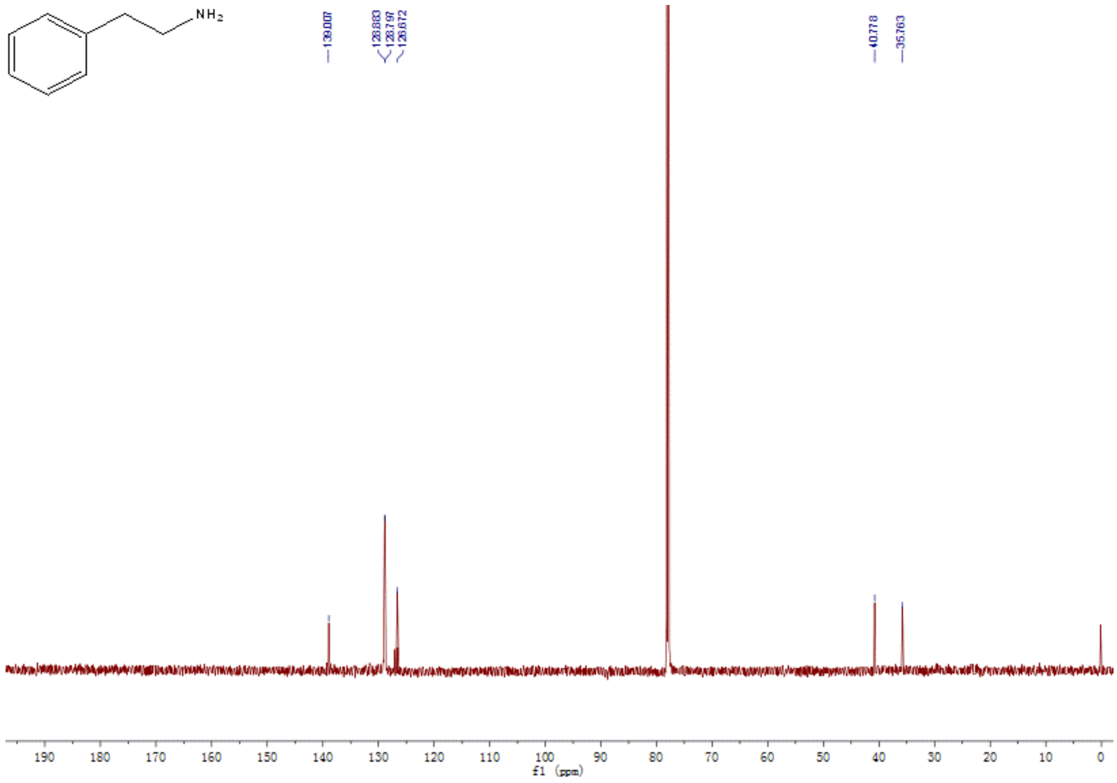

Secondary amines

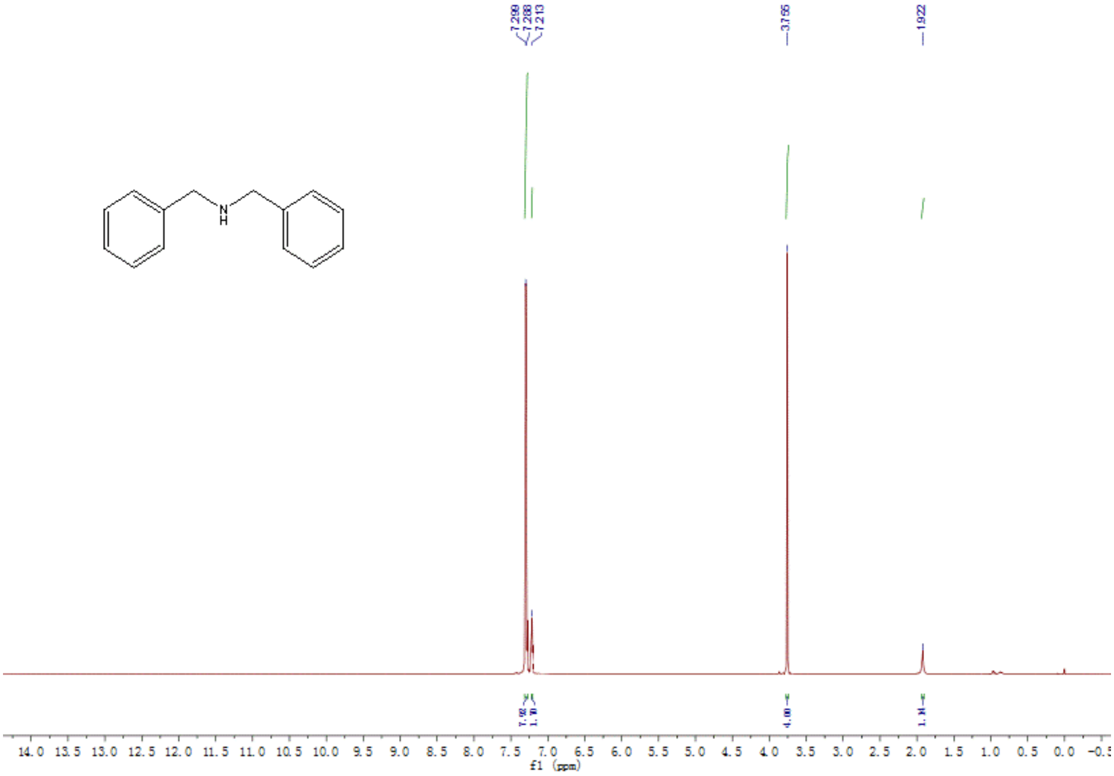

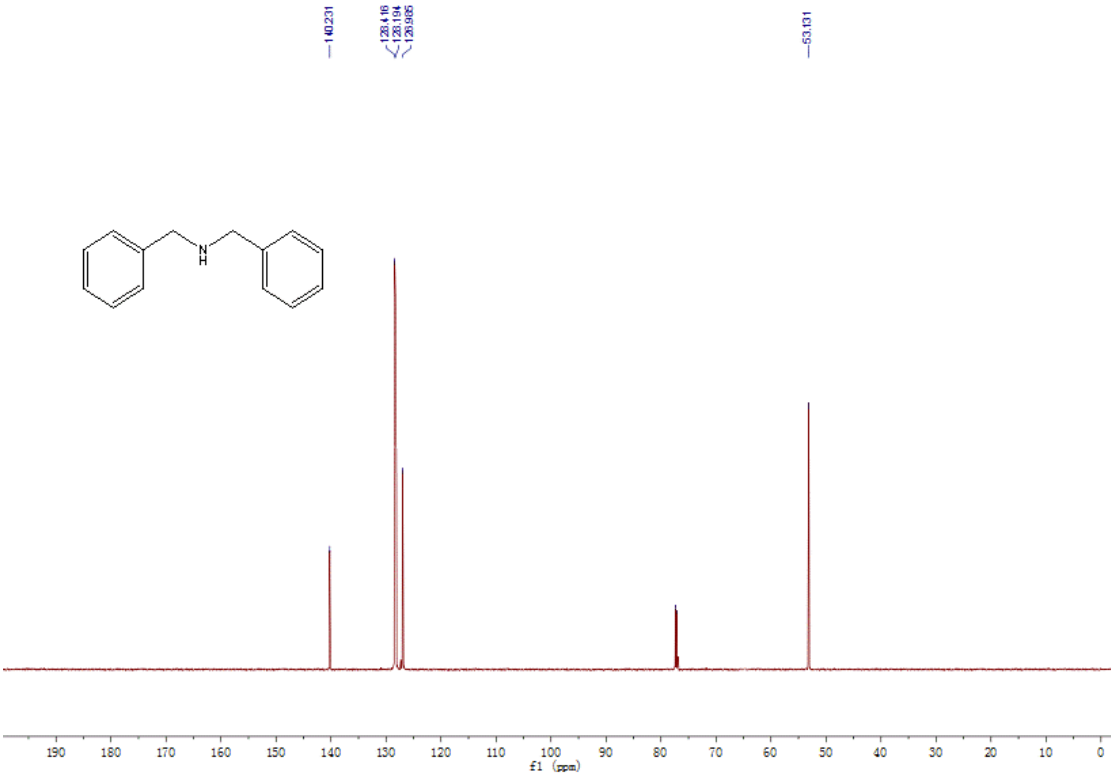

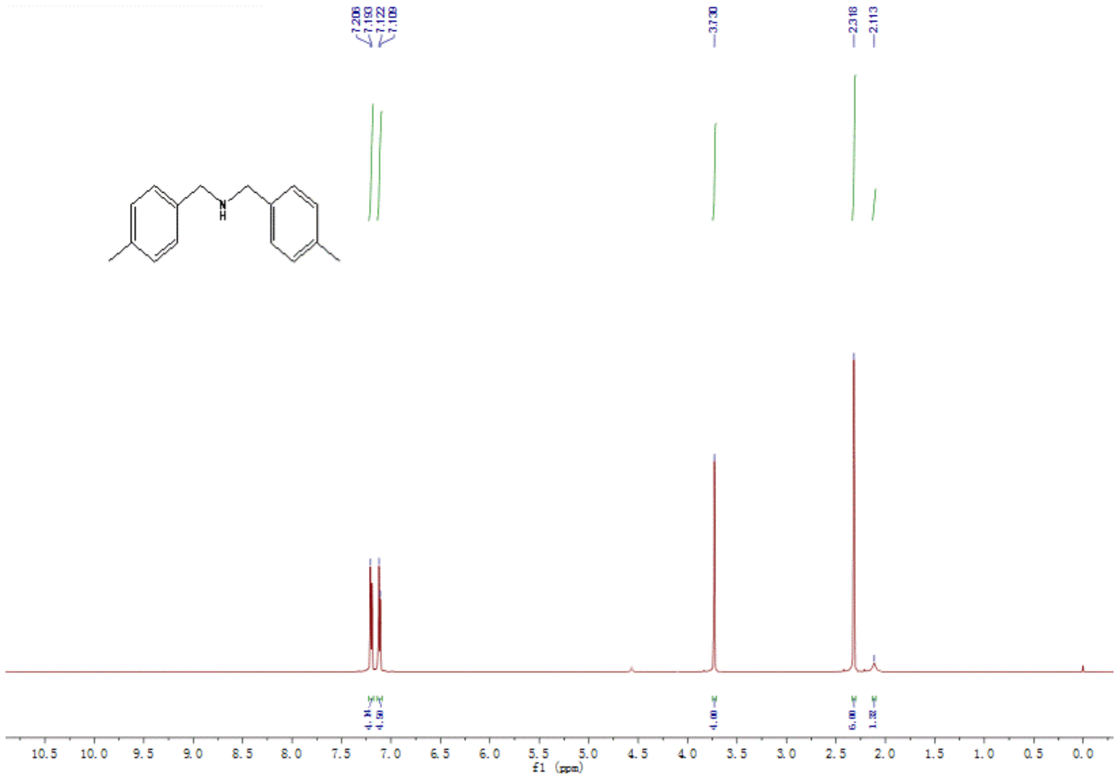
7.206
7.193
7.122
7.109
3.730
2.318
2.113
N
H
f1 (ppm)

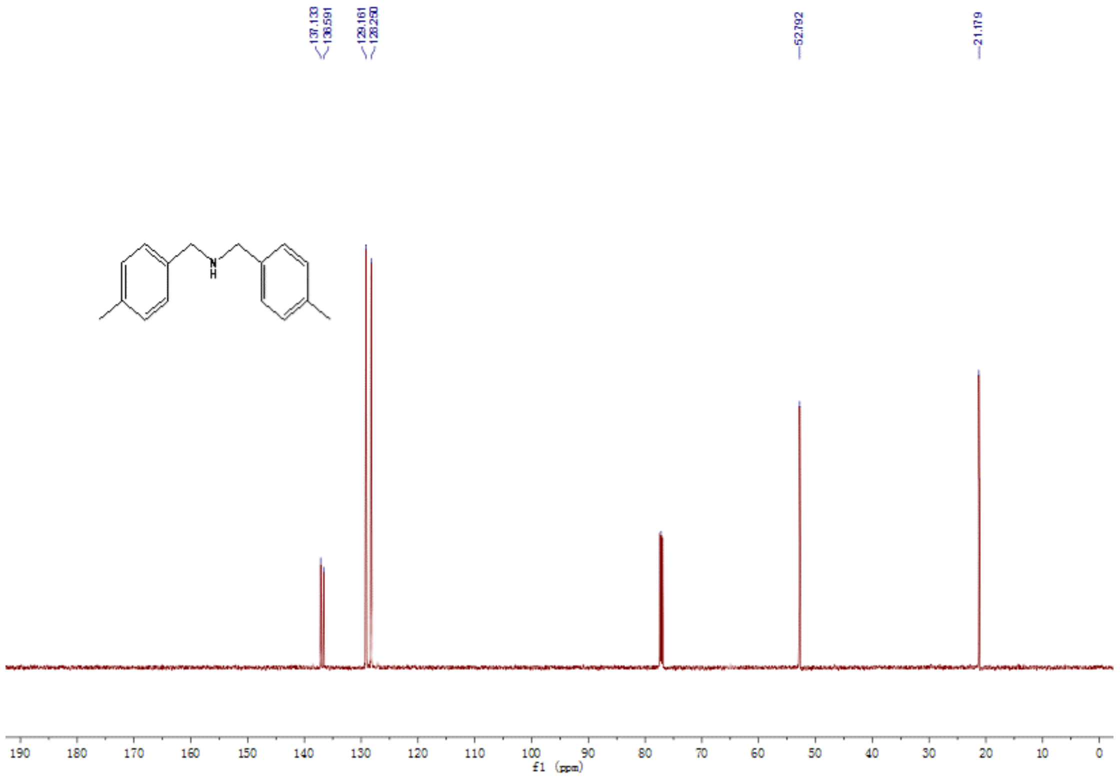
137.133
136.591
129.161
128.250
52.792
21.179
N
H
f1 (ppm)

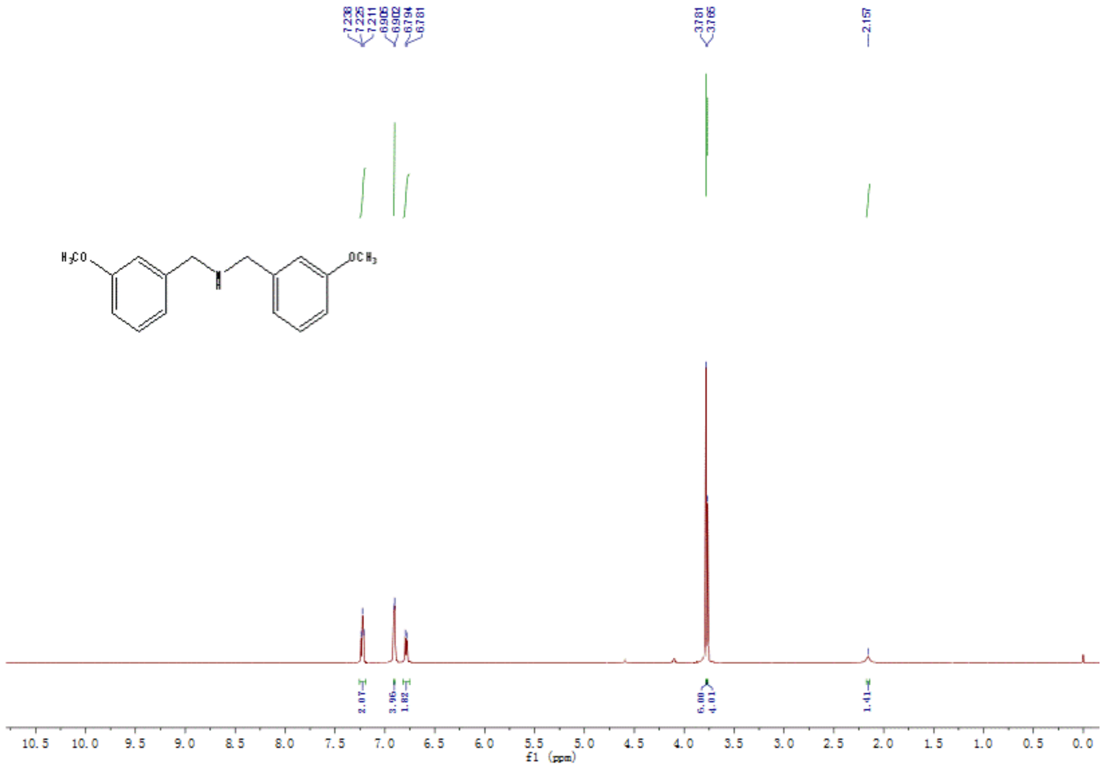
7.238
7.225
7.211
6.905
6.902
6.794
6.781
3.781
3.765
2.157
H3CO
OCH3
N
H
2.07
3.96
1.82
6.00
4.01
1.41
10.5
10.0
9.5
9.0
8.5
8.0
7.5
7.0
6.5
6.0
5.5
5.0
4.5
4.0
3.5
3.0
2.5
2.0
1.5
1.0
0.5
0.0
f1 (ppm)

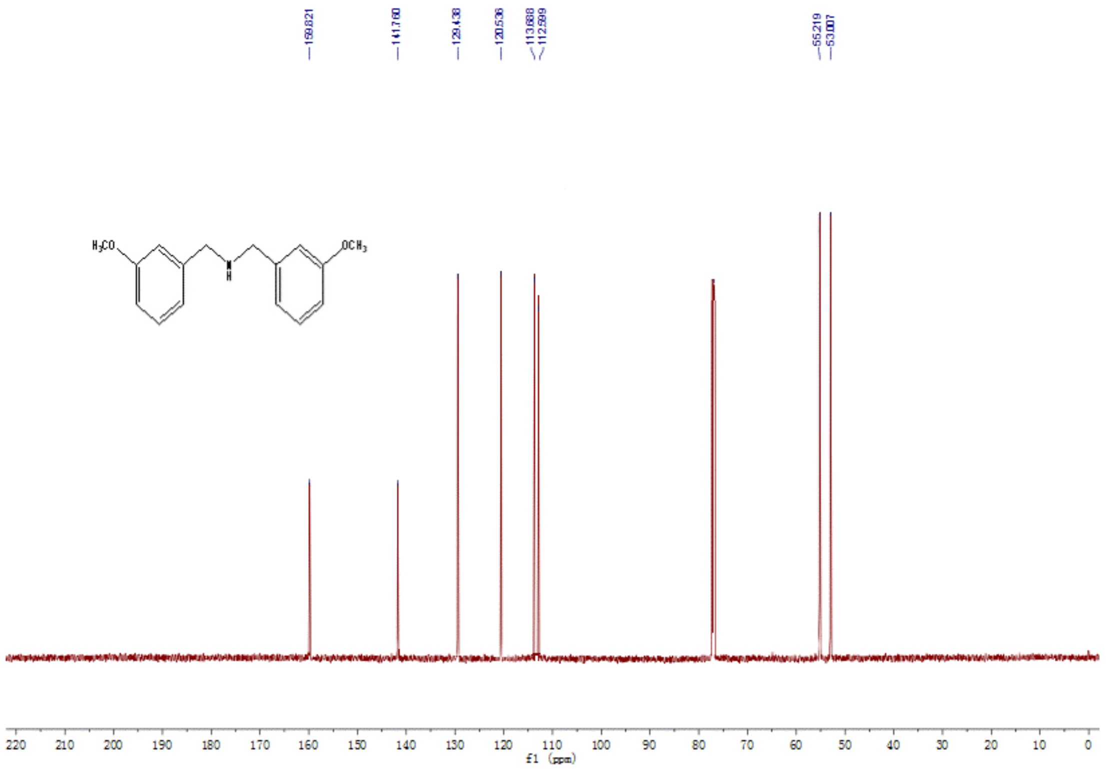
159.821
141.760
129.438
120.536
113.688
112.599
55.219
53.007
H3CO
OCH3
N
H
220
210
200
190
180
170
160
150
140
130
120
110
100
90
80
70
60
50
40
30
20
10
0
f1 (ppm)

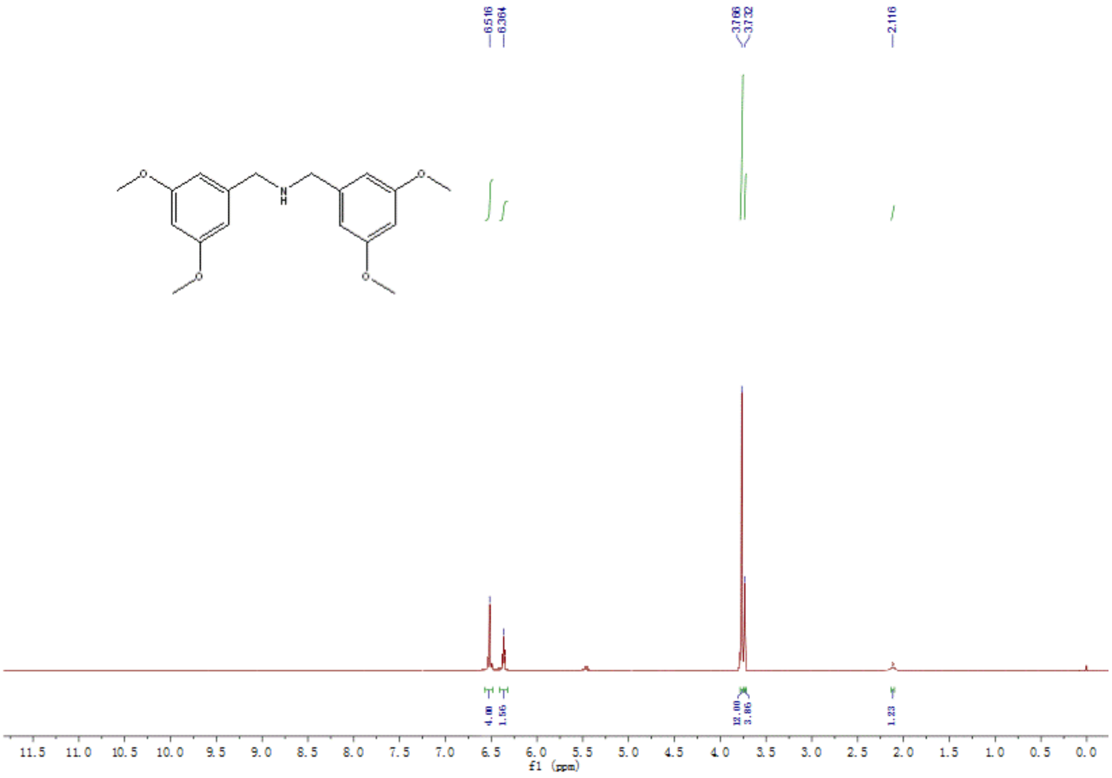
6.516
6.364
3.766
3.732
2.116
4.00
1.56
12.00
3.86
1.23
11.5 11.0 10.5 10.0 9.5 9.0 8.5 8.0 7.5 7.0 6.5 6.0 5.5 5.0 4.5 4.0 3.5 3.0 2.5 2.0 1.5 1.0 0.5 0.0
f1 (ppm)

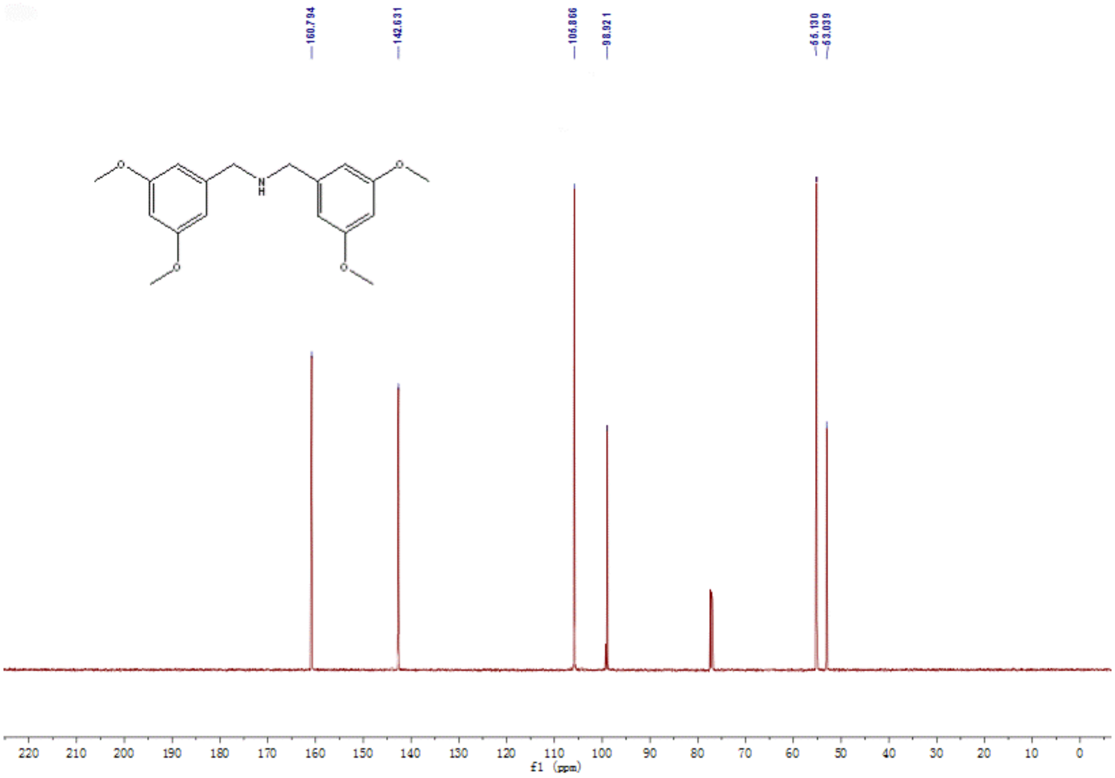
160.794
142.631
105.866
98.921
55.130
53.039
220 210 200 190 180 170 160 150 140 130 120 110 100 90 80 70 60 50 40 30 20 10 0
f1 (ppm)

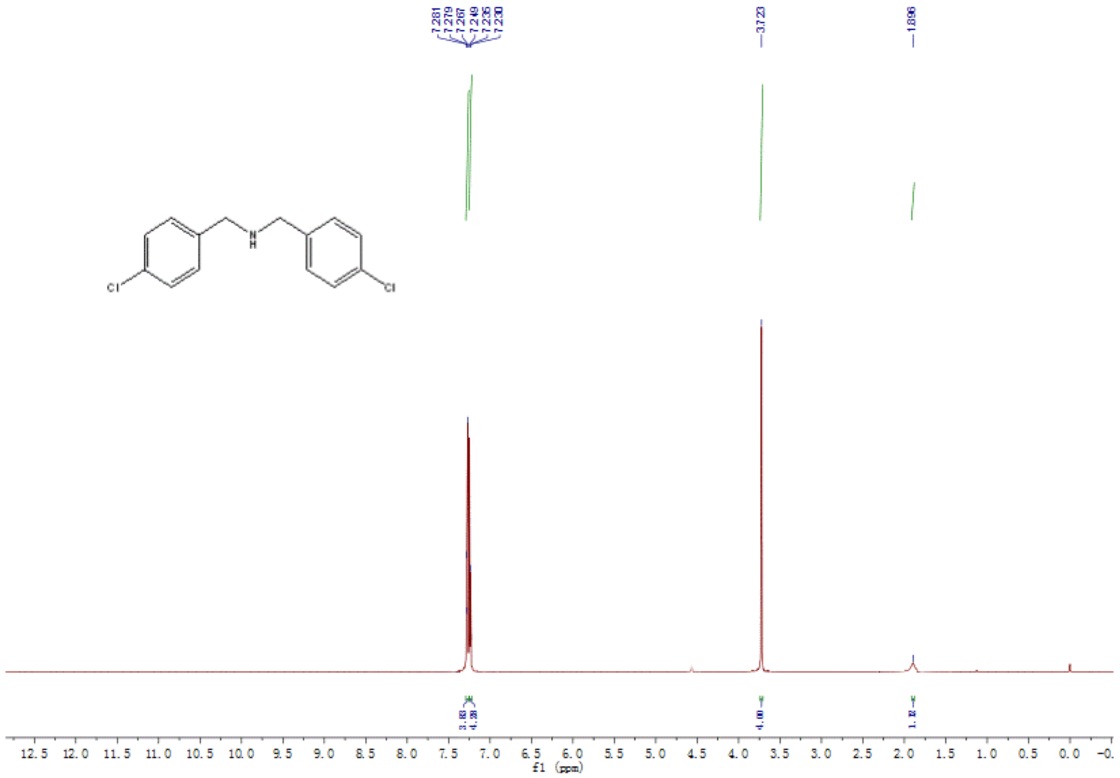
7.281
7.279
7.267
7.249
7.235
7.230
3.723
1.896
Cl
H
N
Cl
3.83
4.39
4.00
1.12
12.5 12.0 11.5 11.0 10.5 10.0 9.5 9.0 8.5 8.0 7.5 7.0 6.5 6.0 5.5 5.0 4.5 4.0 3.5 3.0 2.5 2.0 1.5 1.0 0.5 0.0
f1 (ppm)

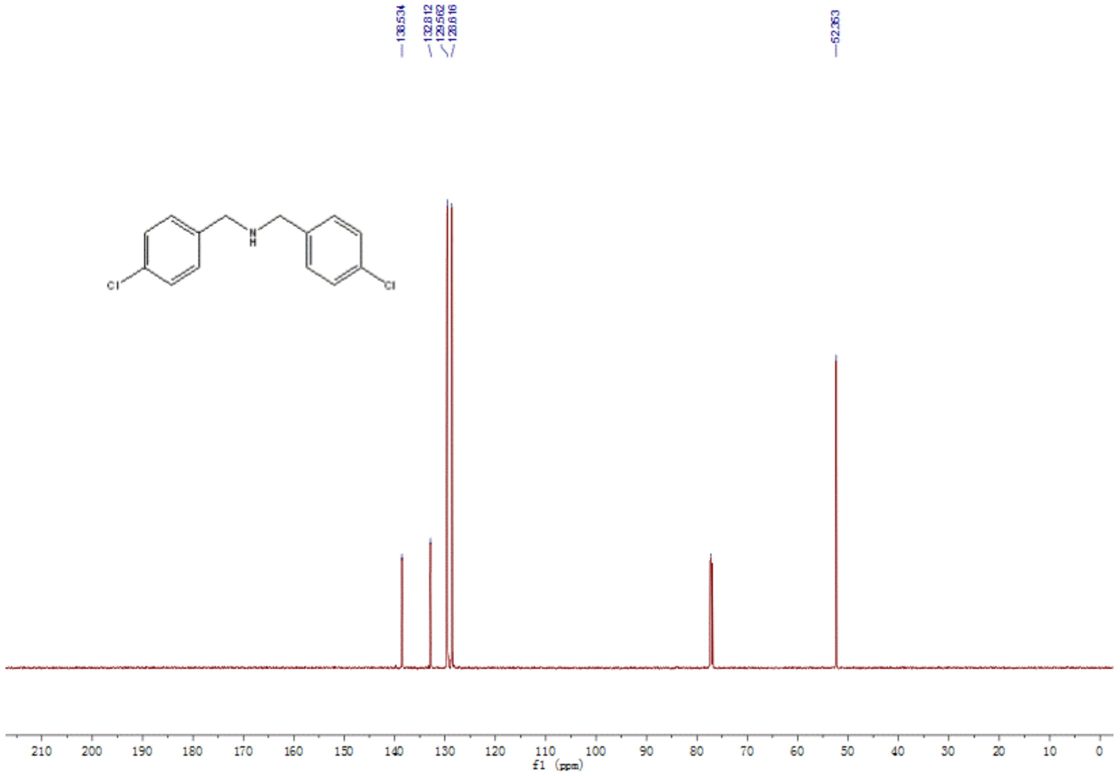
138.534
132.812
129.562
128.616
52.363
Cl
H
N
Cl
210 200 190 180 170 160 150 140 130 120 110 100 90 80 70 60 50 40 30 20 10 0
f1 (ppm)

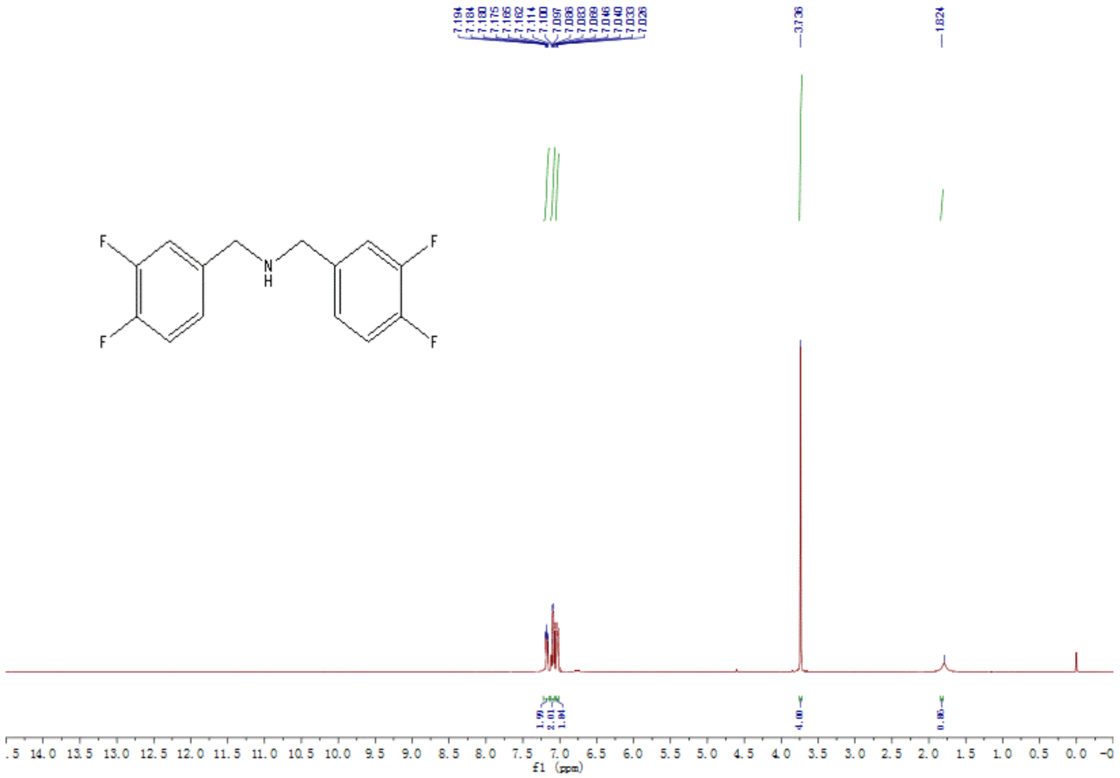
7.194
7.184
7.180
7.175
7.165
7.162
7.114
7.100
7.097
7.086
7.083
7.069
7.046
7.040
7.033
7.026
3.736
1.824
1.99
2.01
1.84
4.00
0.86
f1 (ppm)

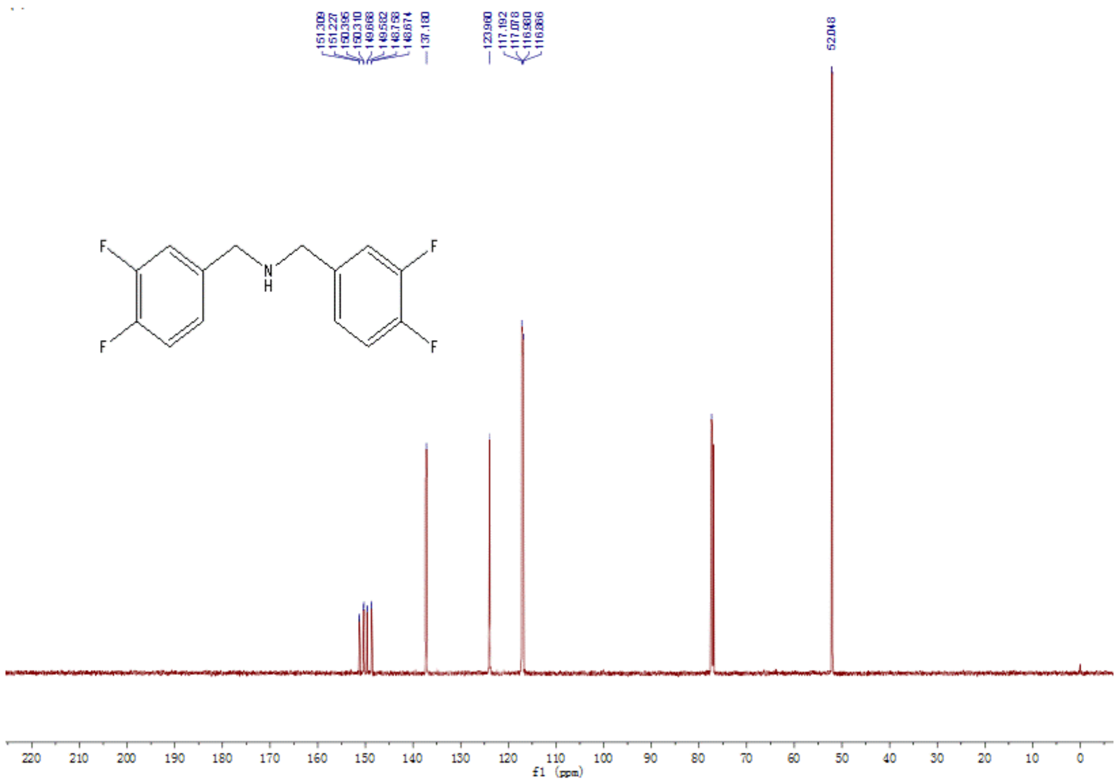
151.309
151.227
150.395
150.310
149.668
149.582
148.758
148.674
137.180
123.960
117.192
117.078
116.980
116.866
52.048
f1 (ppm)

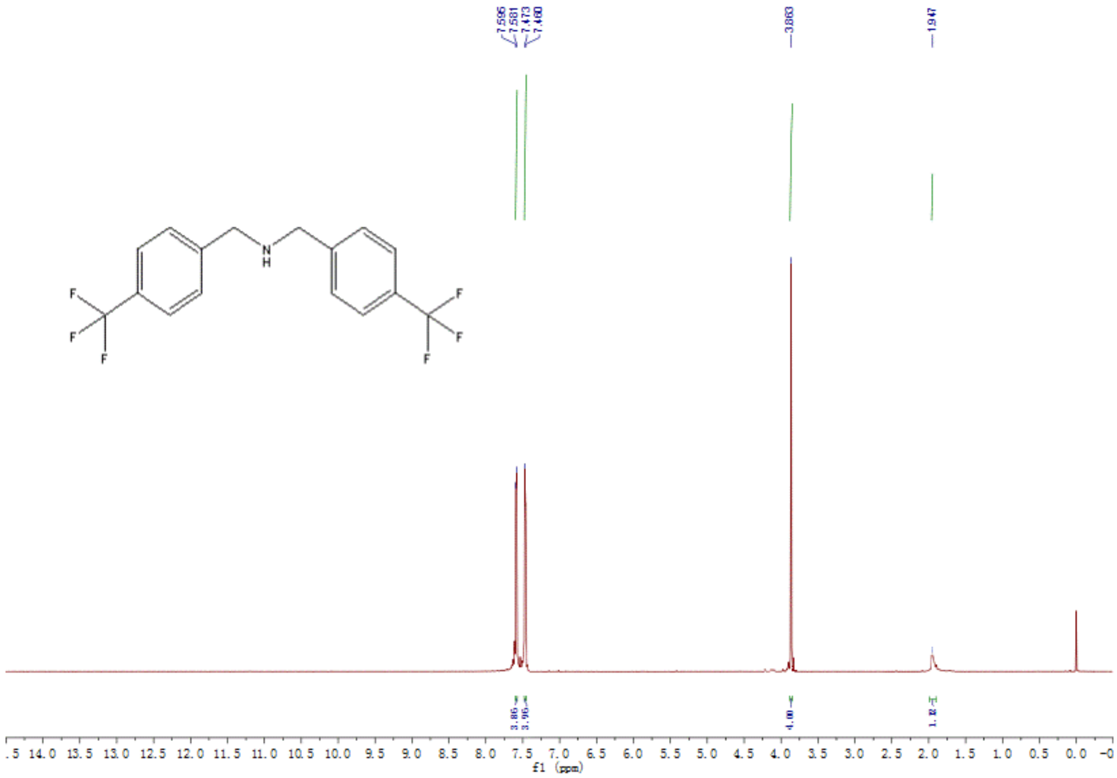
7.595
7.581
7.473
7.460
3.863
1.947
3.86
3.96
4.00
f1 (ppm)

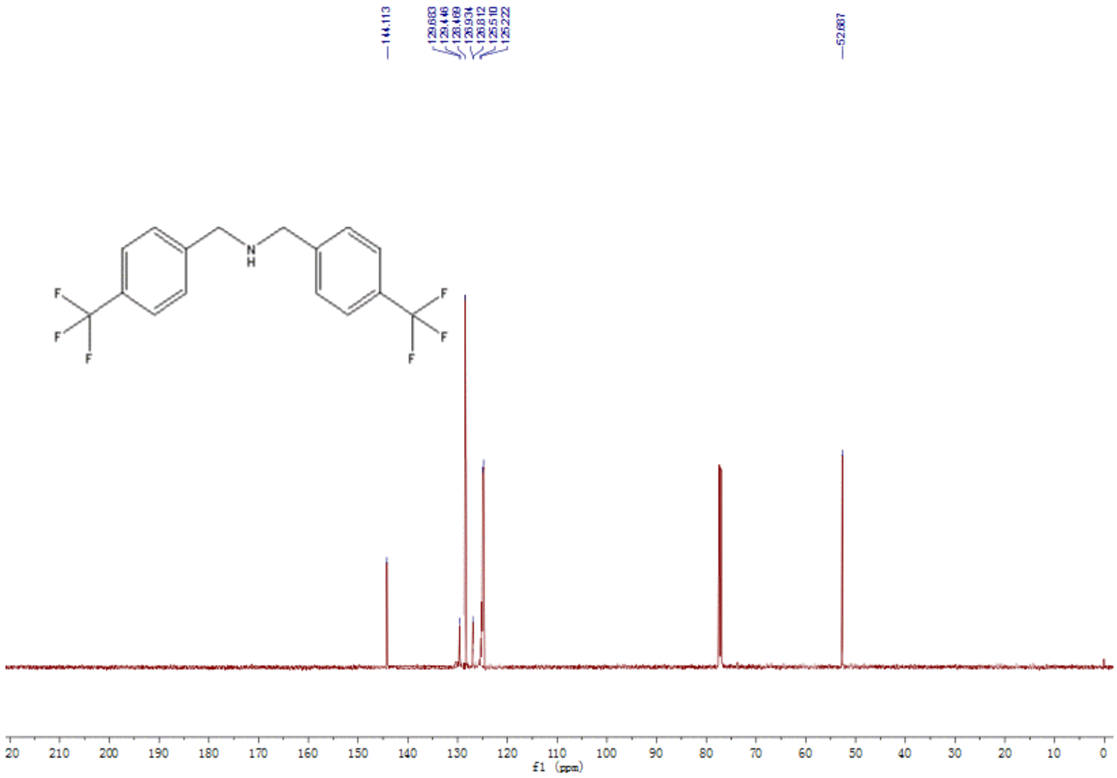
144.113
129.683
129.446
128.489
126.934
126.812
125.510
125.222
52.687
f1 (ppm)

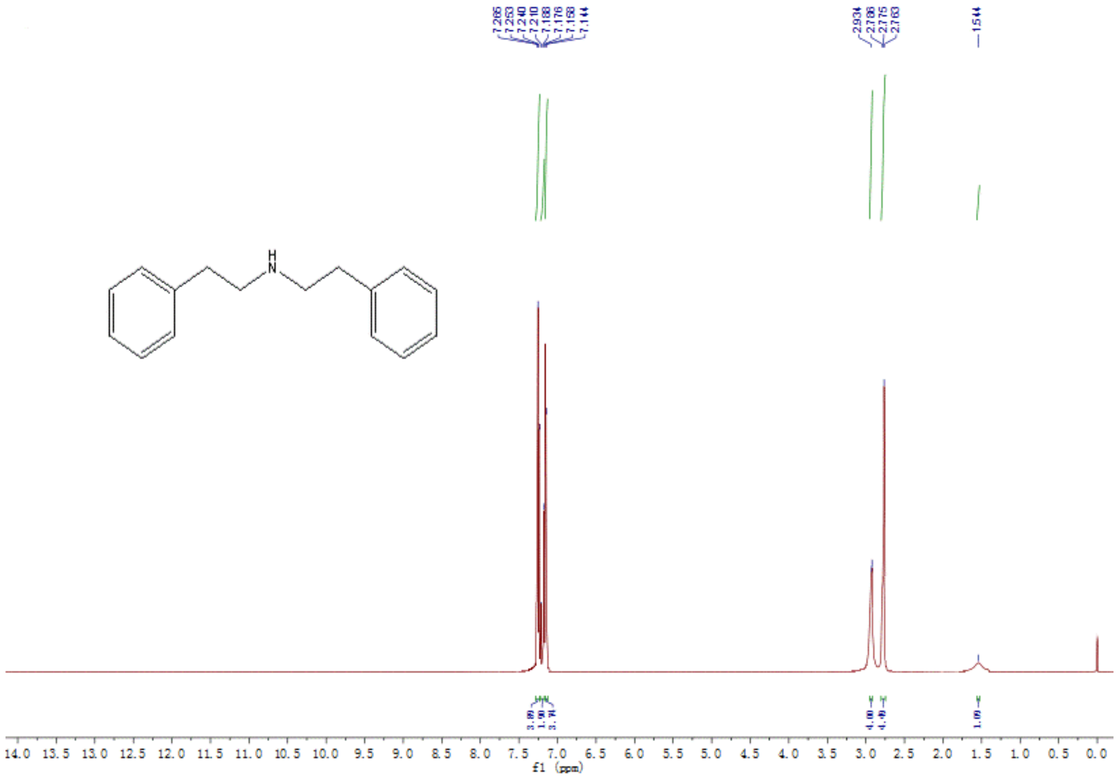

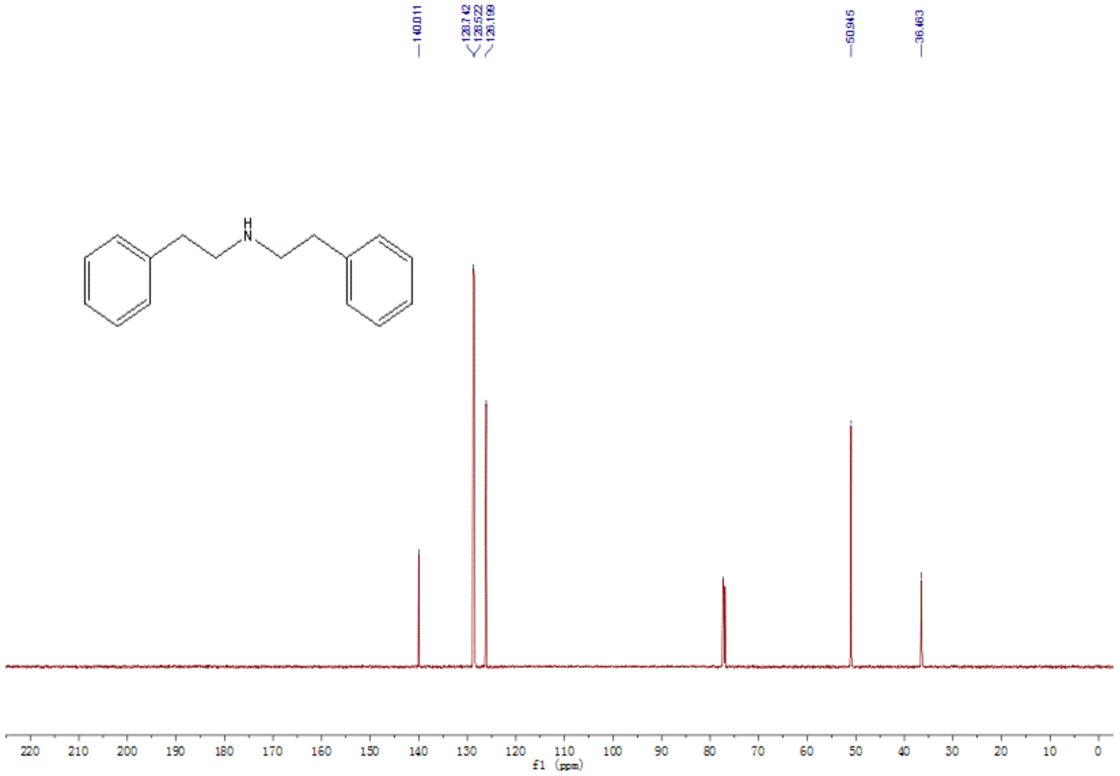

Supplementary reference

1. Dai C, *et al.* Efficient and selective hydrogenation of benzonitrile to benzylamine: improvement on catalytic performance and stability in a trickle-bed reactor. *New J. Chem.* **41**, 3758-3765 (2017).

2. McMillan L, *et al.* The application of a supported palladium catalyst for the hydrogenation of aromatic nitriles. *J. Mol. Catal. a-Chem.***411**, 239-246 (2016).

3. Hegedus L, Mathe T. Selective heterogeneous catalytic hydrogenation of nitriles to primary amines in liquid phase Part I. Hydrogenation of benzonitrile over palladium. *Appl. Catal. A* **296**, 209-215 (2005).

4. Dai C, Li Y, Ning C, Zhang W, Wang X, Zhang C. The influence of alumina phases on the performance of Pd/Al2O3 catalyst in selective hydrogenation of benzonitrile to benzylamine. *Appl. Catal. A* **545**, 97-103 (2017).

5. Wang H, et al. Quasi Pd1Ni single-atom surface alloy catalyst enables hydrogenation of nitriles to secondary amines. *Nat. Commun.* **10**, (2019).

6. Greenfield H. HYDROGENATION OF BENZONITRILE TO DIBENZYLAMINE. *Ind. Eng. Chem. Prod. Res.Dev.***15**, 156-158 (1976).

7. Paul H, Basu S, Bhaduri S, Lahiri GK. Platinum carbonyl derived catalysts on inorganic and organic supports: a comparative study. *J. Organomet. Chem.* **689**, 309-316 (2004).

8. Rylander PN, Hasbrouck L, Karpenko I. Coupling reactions of nitriles and anilines during hydrogenation. *Ann. N.Y. Acad. Sci.* **214**, 100-109 (1973).

9. Chatterjee M, Kawanami H, Sato M, Ishizaka T, Yokoyama T, Suzuki T. Hydrogenation of nitrile in supercritical carbon dioxide: a tunable approach to amine selectivity. *Green Chem.* **12**, 87-93 (2010).

10. Ortiz-Cervantes C, Iyanez I, Garcia JJ. Facile preparation of ruthenium nanoparticles with activity in hydrogenation of aliphatic and aromatic nitriles to amines. *J. Phys.Org. Chem.* **25**, 902-907 (2012).

11. Muratsugu S, et al. Formation and nitrile hydrogenation performance of Ru nanoparticles on a K-doped Al_2O_3 surface. *Phys. Chem. Chem. Phys.* **17**, 24791-24802 (2015).

12. Lopez-De Jesus YM, Johnson CE, Monnier JR, Williams CT. Selective Hydrogenation of Benzonitrile by Alumina-Supported Ir-Pd Catalysts. *Top Catal.* **53**, 1132-1137 (2010).

13. Gandhamsetty N, Jeong J, Park J, Park S, Chang S. Boron-Catalyzed Silylative Reduction of Nitriles in Accessing Primary Amines and Imines. *J. Org. Chem.* **80**, 7281-7287 (2015).

14. Tokmic K, Jackson BJ, Salazar A, Woods TJ, Fout AR. Cobalt-Catalyzed and Lewis Acid-Assisted Nitrile Hydrogenation to Primary Amines: A Combined Effort. *J. Am. Chem. Soc.* **139**, 13554-13561 (2017).

15. Dai H, Guan H. Switching the Selectivity of Cobalt-Catalyzed Hydrogenation of Nitriles. *ACS Catal.* **8**, 9125-9130 (2018).

16. Lu S, Wang J, Cao X, Li X, Gu H. Selective synthesis of secondary amines from nitriles using Pt nanowires as a catalyst. *Chem Commun.* **50**, 3512-3515 (2014).

17. He W, et al. Pt-Sn/?-Al_2O_3-Catalyzed Highly Efficient Direct Synthesis of Secondary and Tertiary Amines and Imines. *Chem. Eur. J.* **17**, 13308-13317 (2011).

18. Shao Z, Fu S, Wei M, Zhou S, Liu Q. Mild and Selective Cobalt-Catalyzed Chemodivergent Transfer Hydrogenation of Nitriles. *Angew Chem Int Ed Engl.* **55**, 14653-14657 (2016).
